# Poly(3,4-ethylenedioxythiophene) Nanotube-Decorated Screen-Printed Electrodes with a Ferrocene-Derived Built-in Probe Enables Alpha-Fetoprotein Detection

**DOI:** 10.1021/acs.langmuir.6c00717

**Published:** 2026-05-27

**Authors:** I-Chen Wang, Jayakrishnan Aerathupalathu Janardhanan, Jia-Wei She, Hsiao-hua Yu

**Affiliations:** Smart Organic Materials Laboratory (SOML), Institute of Chemistry, 38017 Academia Sinica, No. 128, Sec. 2, Research Institute Road, Nankang, Taipei City 115201, Taiwan

## Abstract

An easy-to-engineer nanobiosensor electrode platform was developed using commercial screen-printed electrode (SPE) as the sensor substrate, enabling cost-effective detection of alpha-fetoprotein (AFP), a clinically important biomarker of liver cancer. The electrode surface was modified with poly­(EDOT-COOH-*co*-EDOT-EG3) and poly­(EDOT-N_3_) nanotubes via a template-free electropolymerization approach. Incorporation of poly­(EDOT-COOH) enables reactive carboxyl groups useful for covalent conjugation of the AFP antibody (AFP-Ab)—the biomarker detection probe through EDC/Sulfo-NHS coupling chemistry while poly­(EDOT-EG3) imparted antifouling properties to minimize nonspecific biomolecular interactions. In addition, poly­(EDOT-N_3_) introduced to facilitate the conjugation of ethynylferrocene through CuAAC click chemistry, thereby creating a built-in redox probe to bypass the need of a complex external probe, highlighting the key feature of this work. The nanostructure formation from the bilayer of poly­(EDOT-COOH-*co*-EDOT-EG3) and poly­(EDOT-N_3_) was optimized during electrochemical deposition and characterized through scanning electron microscopy (SEM) technique. The modified polymer surfaces were extensively characterized using quartz crystal microbalance (QCM), X-ray photoelectron spectroscopy (XPS), Cyclic voltammetry (CV), and Differential Pulse Voltammetry (DPV) techniques. The SPE nanobiosensor platform successfully detected AFP in phosphate-buffered saline (PBS) using DPV across a wide range of concentrations (1 pg/mL to 1 μg/mL), with a good linearity (*R* ^2^ = 0.9247) and a limit-of-detection (LOD) of 0.603 pg/mL. Furthermore, the SPE nanobiosensor demonstrated reliable stability, reproducibility, and excellent selectivity upon the addition of interfering compounds such as Bovine Serum Albumin (BSA), Tumor Necrosis Factor-α (TNF-α), Neuropeptide-Y (NPY), and Interleukin-6 (IL-6), highlighting its future practical utility to develop healthcare monitoring devices for point-of-care testing (POCT) applications.

## Graphical abstract

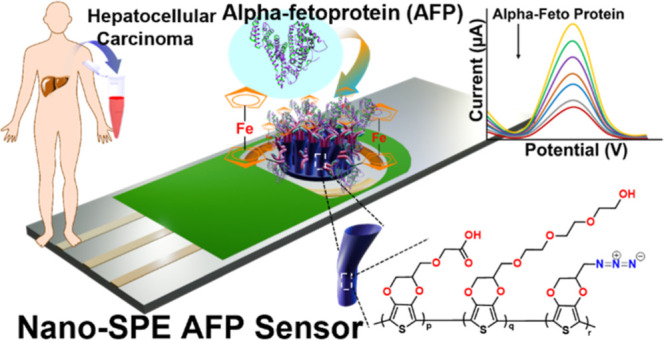

## Introduction

Cancer represents a major public health challenge and remains one of the leading causes of mortality worldwide.^1–3^ Its burden continues to rise, driven by population growth, aging, and increased exposure to environmental and lifestyle-related risk factors.^4–6^ Therefore, accurate, timely, and early detection of various cancers is crucial for improving public health outcomes. Among these malignancies, liver cancer is one of the most prevalent and deadly forms, frequently associated with chronic hepatitis infection or metabolic disorders.^7,8^ Its treatment options are limited. As a result, liver cancer carries a poor prognosis and remains a significant global health burden.^9,10^ Several biomarkers have been investigated for the diagnosis and monitoring of liver cancer, including alpha-fetoprotein (AFP), des-gamma-carboxy prothrombin (DCP, also known as PIVKA-II), and AFP-L3, a more specific glycoform of AFP. In addition, emerging biomarkers such as glypican-3 (GPC3), Golgi protein 73 (GP73), circulating tumor DNA, and various microRNAs have shown potential to improve diagnostic performance.^11–13^ Among these, AFP remains the most extensively studied and widely applied biomarker in clinical practice, owing to its accessibility, cost-effectiveness, and long history of use in both diagnosis and disease monitoring.^14–16^ AFP was selected as the primary biomarker because it has long been established as a clinically important biomarker for hepatocellular carcinoma (HCC) and is also widely recognized in the diagnosis and monitoring of germ cell tumors. Its dual clinical relevance highlights its significance in both hepatic and extrahepatic malignancies.^17–19^ Different methods have been developed to detect AFP, ranging from traditional immunoassays such as enzyme-linked immunosorbent assay (ELISA) and radioimmunoassay (RIA) in the modern platforms including lateral flow immunoassay (LFIA), surface plasmon resonance (SPR), and electrochemical biosensors.^20–24^ However, these methods often have limitations such as low sensitivity, poor selectivity, time-consuming procedures, and high costs.^25,26^ Therefore, it is crucial to develop novel strategies that enable accurate, sensitive, and cost-effective detection of AFP. Among the available platforms, electrochemical biosensors have attracted considerable attention due to their inherent advantages, including excellent selectivity, rapid response, and operational simplicity.^27,28^ In addition, their compatibility with miniaturization and point-of-care testing (POCT) makes them highly promising for clinical applications, particularly in the early detection of liver cancer diseases.^29,30^

Conducting polymers have emerged as an attractive material for electrochemical biosensors because of their excellent electrical conductivity, tunable surface chemistry, and biocompatibility.^31–33^ Among them, poly­(3,4-ethylenedioxythiophene) (PEDOT) derivatives are particularly appealing due to their superior stability, facile functionalization, and ability to provide an efficient interface for biomolecule immobilization.^34–36^ Furthermore, nanostructured PEDOT exhibits significant advantages in sensing, as its enlarged surface area and favorable electronic properties enable improved sensitivity and signal transduction.^37–39^ Template-free electropolymerization further offers a simple and efficient method to produce nanostructured surfaces without requiring complex fabrication procedures.^40,41^ These properties make nanoassembled PEDOT materials highly suitable candidates for enhancing the sensitivity, selectivity, and overall performance to design nanobiosensors for diverse biomarkers detection.

Ferrocene (Fc) is one of the interesting redox probes used in many electrochemical sensor platforms.^42–44^ It has promising redox properties with reversible switching of Fe (II)/Fe (III) at relatively lower potential, a promising advantage suitable to operate in an aqueous environment.^45,46^ However, the stability of ferrocene probe in solution may differ over time, leading to signal instability and inaccuracy in measurements, a major concern while integrating electrochemical sensors into wearable POCT device architectures. In addition, repeated measurements demand restoration of the mediator solution, which might damper sensor reuseability. To simplify the detection procedure, the concept of the “built-in redox probe” to engineer electrochemical sensors has gained increasing attention, as it allows electron shuttle between the electrode surface and the biorecognition events, eliminating the requirement for external redox mediators.^47–49^ When combined with PEDOT-derived nanostructures, this approach not only simplifies sensor architecture but also provides a highly conductive interface that enable enhanced sensitivity, operational stability, and sensor reuseability—an inevitable requirement for developing wearable biosensor platforms. Considering these promising features, we have designed a nanobiosensor platform with a built-in redox probe derived from ferrocene through click chemistry owing to its simplicity and high efficiency of covalent bonds with mild conditions. The ferrocene probe was “clicked” onto the sensor platform through azide-functionalized PEDOT and ethynylferrocene. This design enables analyte detection without the need for external reference probe such as K_4_Fe­(CN)_6_, thereby simplifying the detection process and reducing interference from extraneous redox species.

This paper highlights our design of a unique SPE-electrochemical biosensor platform based on PEDOT-derived nanostructures with a built-in redox probe to detect AFP biomarkers (Figure 1). The sensor design comprises of diverse molecular architectures of PEDOTs including PEDOT-COOH, PEDOT-EG3, and PEDOT-N_3_ functionalities to create tunable interfaces for biomolecule immobilization while simultaneously enhancing biocompatibility. Prior to the target analyte detection, the SPE electrode platform was modified with nanostructured PEDOT derivatives bearing diverse functional groups. Poly­(EDOT-COOH-*co*-EDOT-EG3) nanostructures were initially deposited on the SPE electrode through template-free electropolymerization technique. The polymer nanostructure formation was optimized by adjusting the electropolymerization parameters. The second layer of polymer nanostructures were deposited from poly­(EDOT-N_3_), offering conjugation site for the ferrocene redox probe. The nanostructures derived from poly­(EDOT-N_3_) were also optimized prior to the click reaction. The key design of the sensor platform highlights the incorporation of a built-in probe derived from ferrocene through copper-catalyzed Azide-Alkyne Cycloaddition (CuAAC) reaction. The successful covalent immobilization of the ferrocene probe through the click reaction was confirmed through cyclic voltammetry and X-ray photoelectron spectroscopy. Following that, the detection probe immobilization was successfully performed with AFP-Ab onto the COOH groups of the poly­(EDOT-COOH) layer through the amide bond formation by EDC/Sulfo-NHS coupling chemistry. The successful immobilization of the antibody onto the SPE electrode platform was confirmed by QCM technique, electrochemical and spectroscopic techniques. The ready-to-use nanobiosensor platform was used to detect target analyte using Differential pulse voltammetry (DPV) technique. AFP concentrations ranging from 1 pg/mL to 1 μg/mL in PBS buffer were tested using DPV and the sensor platform exhibited an excellent linearity (*R* ^2^ = 0.9247) and LOD of 0.603 pg/mL. Furthermore, the stability, shelf life, and reproducibility of the SPE nanobiosensor was examined in detail. Our nano-SPE sensor platform showed promising shelf life of 10 days as well as excellent selectivity upon the addition of interfering compounds such as Bovine Serum Albumin (BSA), Tumor Necrosis Factor-α (TNF-α), Neuropeptide-Y (NPY), and Interleukin-6 (IL-6), highlighting the exploration of the nano-SPE sensor platform for wearable devices and POCT applications.

**Figure 1:**
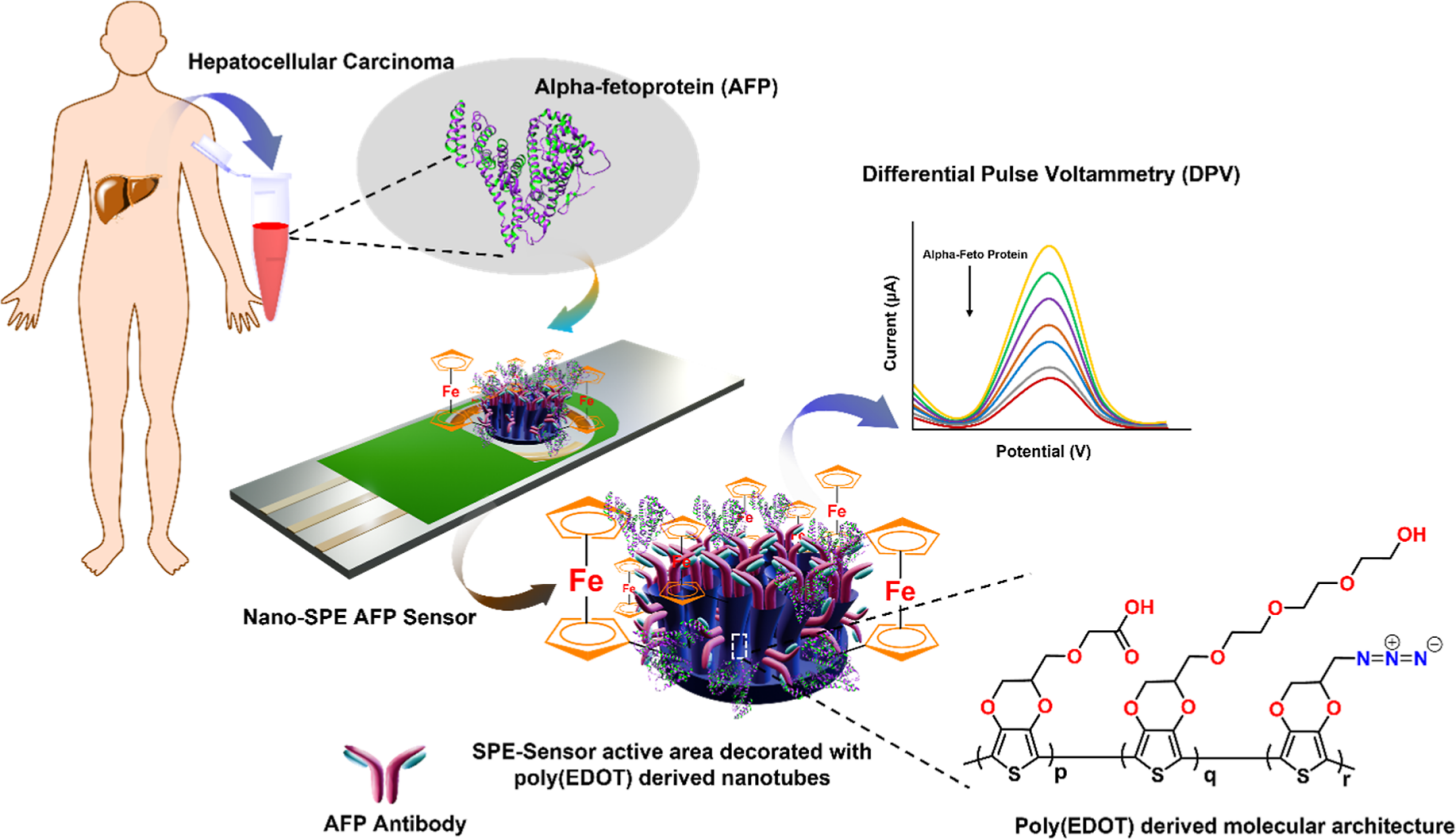
Schematic illustration of the design of the nano-SPE sensor platform for AFP detection. The SPE electrode active area was decorated with poly­(EDOT-COOH-*co*-EDOT-EG3) nanotubes by the template-free electropolymerization method. Additionally, the second layer of the polymer was deposited on the nanotubes from poly­(EDOT-N_3_). The molecular architecture enables click chemistry-assisted conjugation of the ferrocene scaffold which served as a built-in probe, while –COOH side chain offers an easy conjugation site to immobilize the AFP antibody through EDC/Sulfo-NHS coupling chemistry. Ethylene glycol (EG3) groups are best known to work as antifouling agents to reduce nonspecific interaction in the biological environments.

## Experimental Section

### Materials and Methods

EDOT–OH (95+%; Angene Chemicals), tetrabutylammonium perchlorate (TBAP), 1-(3-(Dimethylamino)­propyl)-3-ethylcarbodiimide Hydrochloride (EDC–HCl, 98%), and Sodium ascorbate (98%) were purchased from TCI Japan. *N*-hydroxysulfosuccinimide sodium salt (Sulfo-NHS, 98%, Combi-Blocks), Copper­(II) sulfate pentahydrate (99% DUKSAN), *N*,*N*-dimethylformamide (DMF, 99.8% anhydrous, Sigma-Aldrich), and Dichloromethane (DCM, Thermo Fisher Scientific) were of analytical grade and used as received. Ethynylferrocene was purchased from Combi-Blocks. Phosphate-buffered saline (PBS, 1x, pH 7.4, Gibco, Thermo Fisher Scientific) was employed as a standard for the QCM studies and also served as the electrolyte in the differential pulse voltammetry (DPV) measurements. Gold-coated Screen-Printed electrodes (SPE-250 AT) with a geometric area of 0.11 cm^2^ and Cable Connectors for SPE were purchased from Metrohm. Surface morphologies of the polymer films formed on SPE electrodes were characterized using a Zeiss-Ultra Plus field-emission scanning electron microscope operated at an accelerating voltage of 30 KeV and a working distance of 50 mm. Electrochemical analyses were performed using a potentiostat (PGSTAT128N, Autolab, Metrohm Inc.). TNF-α, Interleukin-6 (IL-6), and Alpha-Fetoprotein antibody (AFP-Ab) were purchased from Sino Biological. Alpha-Fetoprotein (AFP) was purchased from AbboMax. Neuropeptide-Y (NPY) and Bovine serum albumin (BSA) were purchased from Sigma-Aldrich.

### Synthesis of Monomers

The monomers such as EDOT–COOH and EDOT-EG3 were synthesized and characterized according to our previous reports.^37^ The details of the synthesis of EDOT-N_3_ was shown in the Supporting Information.

### Template-Free Electropolymerization to Engineer Nanostructures on SPE Electrodes

Gold-coated SPE electrodes were modified with nanostructures of copolymers engineered from EDOT-COOH, EDOT-EG3, and EDOT-N_3_ via a template-free electropolymerization technique. The electropolymerization was carried out using a potentiostat (PGSTAT128N, Autolab, Metrohm Inc.). We performed two electropolymerization processes separately to modify the electrode surface with polymer nanostructures to design the sensor platform, as detailed below.

### Nanostructure Engineering of EDOT-COOH-*co*-EDOT-EG3 Copolymers on the SPE Electrode Platform

Commercially available Au-SPE electrodes were used to engineer PEDOT derived nanobiosensor platforms. Template-free electropolymerization under varying conditions was employed to construct the nanostructured, first layer of poly­(EDOT-COOH-*co*-EDOT-EG3) on the SPE surface. For this purpose, monomer solutions of EDOT-COOH (5 mM) and EDOT–EG3 (5 mM) in dichloromethane (DCM), with tetrabutylammonium perchlorate (TBAP) (100 mM) as the supporting electrolyte, were prepared. A constant potential of +1.2 V (vs Ag/Ag^+^) was subsequently applied for 90 s at 0–2 °C to deposit polymer nanostructures onto the SPE electrodes after performing optimization experiments to produce an ideal sensor platform decorated with poly­(EDOT-COOH-*co*-EDOT-EG3) polymer nanostructures. A constant charge (Q) was applied during all electropolymerization experiments to ensure that all the SPE electrodes were prepared identically. After polymerization, the electrodes were washed with acetonitrile, followed by double-distilled DI water to remove the residual, unreactive supporting electrolyte and unpolymerized monomers.

### Surface Modification of poly­(EDOT-COOH-*co*-EDOT-EG3)-Coated SPE Electrodes with poly­(EDOT-N_3_)

After the successful engineering of the SPE nanoelectrode with a first layer of poly­(EDOT-COOH-*co*-EDOT-EG3) nanotubes, the electrode surface was further modified with poly­(EDOT-N_3_) through a template-free electropolymerization method using the EDOT-N_3_ monomer. For that, 10 mM EDOT-N_3_ was dissolved in DCM containing 100 mM TBAP as the supporting electrolyte. After that, a constant potential of +1.2 V (vs Ag/Ag^+^) was applied for 60 s at room temperature. To ensure the uniformity of all SPE electrodes, we set a constant charge (Q) for all the electropolymerization experiments after optimization experiments to tune the electrode surface morphology. Following the polymerization process, the electrodes were washed with acetonitrile, subsequently with double-distilled DI water to remove the residual supporting electrolyte and unreacted monomers.

### Post-functionalization of the poly­(EDOT-N_3_)-Coated Nano-SPE Electrode Surface with Ethynylferrocene by Click Chemistry

In order to anchor ethynylferrocene on the nano-SPE electrode surface decorated with poly­(EDOT-N_3_) having azide functional groups, a classical click reaction was performed as follows. At first, the polymer nanotube-decorated SPE electrode platform was immersed in DMF/water (2:1) solution containing ethynylferrocene (0.1M), CuSO_4_·5H_2_O (0.1M), and sodium ascorbate (0.1M) at room temperature overnight. Following this, the SPE electrodes were thoroughly washed with acetonitrile and double-distilled DI water to remove unreacted reagents from the electrode surface. The successful immobilization of ferrocene-derived built-in probe poly­(EDOT-Fc) was confirmed through spectroscopic and electrochemical characterization.

### Antibody Conjugation on the Surface of Nano-SPE Electrodes

The nano-SPE electrode platform, consisting of poly­(EDOT-COOH-*co*-EDOT-EG3) and poly­(EDOT-Fc) nanotubes was further modified with monoclonal alpha-fetoprotein antibody (AFP-Ab) through EDC/Sulfo-NHS coupling chemistry. The antibody serves as the probe for detecting the target analyte. For that, the nanotube-decorated SPE electrode (nano-SPE) was soaked in 0.4 M EDC and 0.1 M Sulfo-NHS in PBS (1×, pH 7.4) for 6 h to activate the carboxylic acid from poly­(EDOT-COOH-*co*-EDOT-EG3) present on the electrode surface. After activation, the electrode was washed with PBS buffer to remove residual, unreacted EDC/Sulfo-NHS reagents. The AFP antibody, dissolved in PBS buffer at a concentration of 100 μg/mL, was dropped onto the electrode to fully cover the polymer area and incubated overnight at 4 °C to form amide bonds between carboxylic acids from the layer of poly­(EDOT-COOH-*co*-EDOT-EG3) and the amine groups of the antibody. After successful conjugation, the nano-SPE electrode was washed with PBS buffer to remove any unbound AFP antibody from the electrode surface. The ready-to-use nano-SPE sensor platform was subsequently used for analyte detection using the differential pulse voltammetry technique.

### Electrochemical Detection of AFP Using the Nano-SPE Sensor Platform

All electrochemical measurements, including Differential Pulse Voltammetry (DPV) and Cyclic Voltammetry (CV), were performed using a potentiostat (PGSTAT128N, Autolab, Metrohm Inc.). The electrochemical detection methods were employed to investigate the interaction of different concentrations of the AFP analyte with newly developed nano-SPE electrodes. The electrodes were treated with different concentrations of AFP analyte in PBS (1x, pH = 7.4) for 15 minutes before measurement to allow antigen–antibody binding. The electrodes were then washed with the same PBS buffer before each experiment. CV was conducted from −0.6 V to +0.8 V at a scan rate of 0.1 V s^–1^ and DPV was performed from 0 V to +0.6 V at a scan rate of 0.01 V s^–1^ with an amplitude of 50 mV. Each experiment was repeated at least 3 times to ensure the reproducibility of the results. The AFP analyte was prepared at concentrations ranging from 1 pg/mL to 1 μg/mL in PBS buffer (1 x, pH = 7.4).

### Shelf Life, Reproducibility, and Repeatability Studies of the Nano-SPE Sensor

All the DPV measurements for shelf life, reproducibility, and repeatability studies were performed by applying a potential from 0 V to +0.6 V (vs Ag). The shelf life of the nano-SPE electrode was evaluated by measuring DPV currents of the nanoelectrode platform incubated with 10 ng/mL AFP solution for 15 minutes. After incubation the electrode was washed with PBS buffer (1x, pH = 7.4) to remove any unbound analytes. When not in use, the electrodes were stored at 4 °C. The measurements were continued for many days until a significant drop in current observed from the DPV measurements. The interelectrode reproducibility was investigated by measuring the DPV current from identically fabricated four electrodes after incubating 10 ng/mL AFP solution for 15 minutes and subsequent PBS wash. For each electrode, the measurements were repeated minimum three times to ensure the consistency of measurements. Similarly, the intraelectrode repeatability was investigated by measuring the current from the nano-SPE electrode after incubating 10 ng/mL AFP solution for 15 minutes and measuring the DPV current three times. The batch-to-batch reproducibility of the nano-SPE electrode platform was examined by fabricating different batches of the nano-SPE electrode identically in a time interval of one, two, and three days. One electrode from each batch was incubated with 10 ng/mL AFP solution for 15 minutes and the current was measured using the DPV method. The DPV measurements were taken for minimum three times for each electrode to ensure the consistency in the measurements.

## Results and Discussion

The construction of PEDOT-derived nanostructures and the functionalization with ethynylferrocene on SPE surfaces through azide-alkyne click chemistry were our main objectives in the design of a novel nanobiosensor platform. Following the conjugation with the AFP antibody (AFP-Ab) as the sensor probe, the target analyte could be directly detected in PBS buffer without the need for external redox probes, owing to the distinctive electrochemical signal of the built-in ferrocene redox probe onto the nanosensor platform. The overall process used to engineer the nano-SPE AFP sensor platform is illustrated in Figure 2. In order to simplify and accelerate the fabrication process, poly­(EDOT-COOH-*co*-EDOT-EG3) and poly­(EDOT-N_3_) nanostructures were constructed on the SPE surface using a template-free electropolymerization method described in the experimental section. Our previous extensive investigations and other research group’s findings show that the DCM/TBAP solvent-supporting electrolyte system is a promising option for the template-free electropolymerization method to design diverse nanostructures of poly­(EDOT) derivatives particularly due to excellent solubility of EDOT monomers, high-quality of the poly­(EDOT) film as well as the nanostructure formation through fine-tuning.^39^ ^,^ ^40^ This method is straightforward to perform and requires significantly less time and minimal amount of monomers, without the need for complex reaction procedures and longer reaction time. Our molecular design strategy to append different conjugation handles such as –COOH and –N_3_ functional groups for the antibody and built-in ferrocene probe conjugation explores diverse and simple coupling chemistry methods. The utility of molecular design is illustrated in Figure 3A. The correlation between morphological metrics with electrochemical response and optimization of nanostructure morphology during the electropolymerization process was the crucial step to engineer the novel nano-SPE electrode platform. Earlier studies from our research group have extensively investigated the formation of different nanostructures, the changes in the structural parameters as well as the morphological metrics according to the polymerization deposition time, monomers’ feed ratio, polymerization temperature, applied potential, and duration of the polymerization. To ensure the formation of homogeneous and uniform nanostructures on the electrode surface, the electropolymerization conditions were optimized by adjusting parameters like applied potential and time, prior to the immobilization of the detection probe.

**Figure 2:**
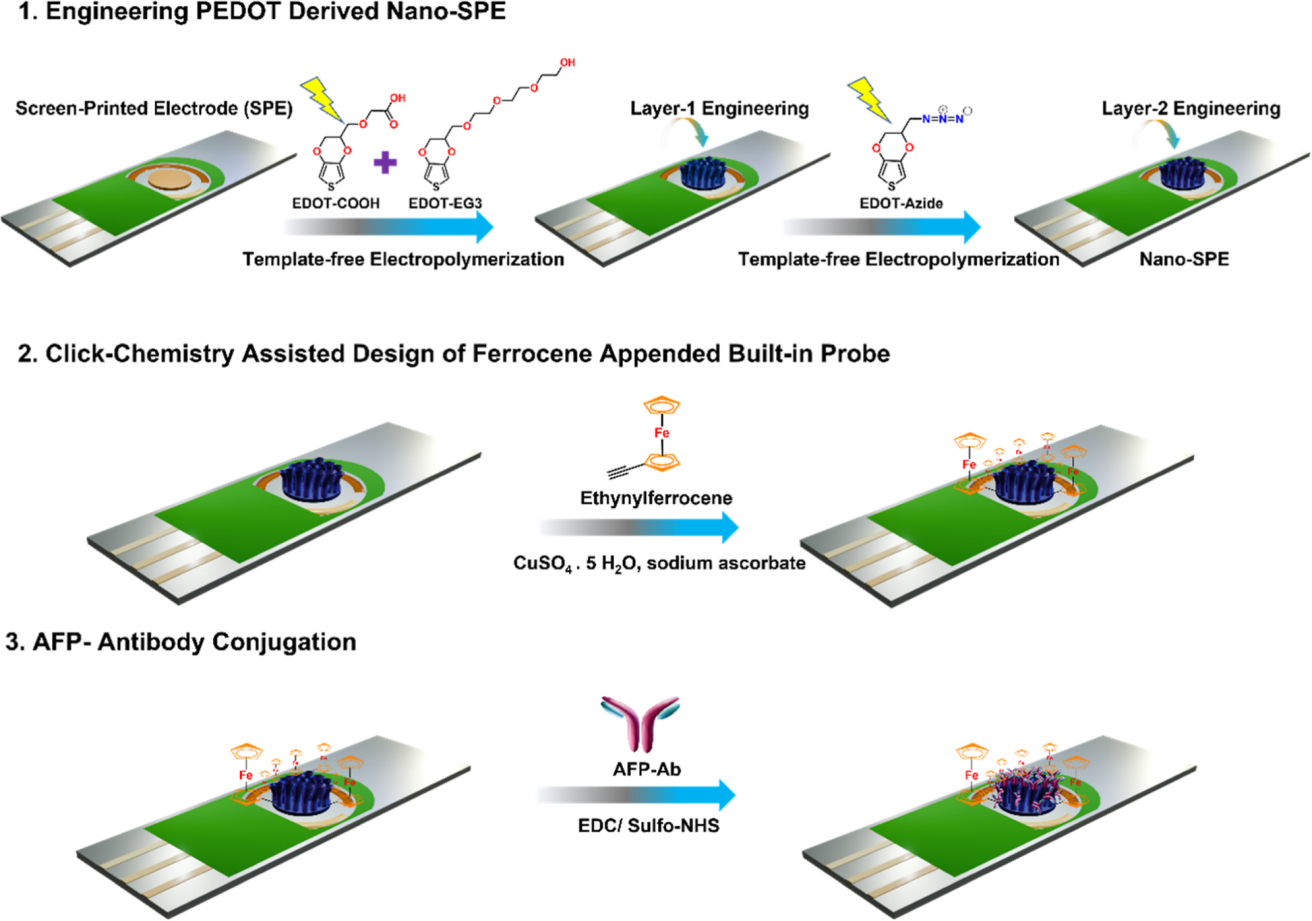
Engineering of the nano-SPE sensor platform with the built-in Ferrocene probe. (1) Commercial Au-SPE electrode was modified with nanotubes of P-NT-COOH/EG3 and P-NT-N_3_ using the template-free electropolymerization method with optimized conditions. (2) The polymer nanotubes on the SPE electrode platform were modified with ethynylferrocene through copper-catalyzed click reaction. The cycloaddition reaction between ethynylferrocene and the azide group from the nanotubes enables the successful conjugation of the ferrocene probe which serves as the built-in probe on the sensor surface. (3) Final-stage of the nano-SPE sensor platform. AFP-Ab was conjugated on the sensor platform to detect target analyte. The EDC-Sulfo-NHS chemistry utilized to bond AFP-Ab on the –COOH handle on the nanotube surface.

**Figure 3:**
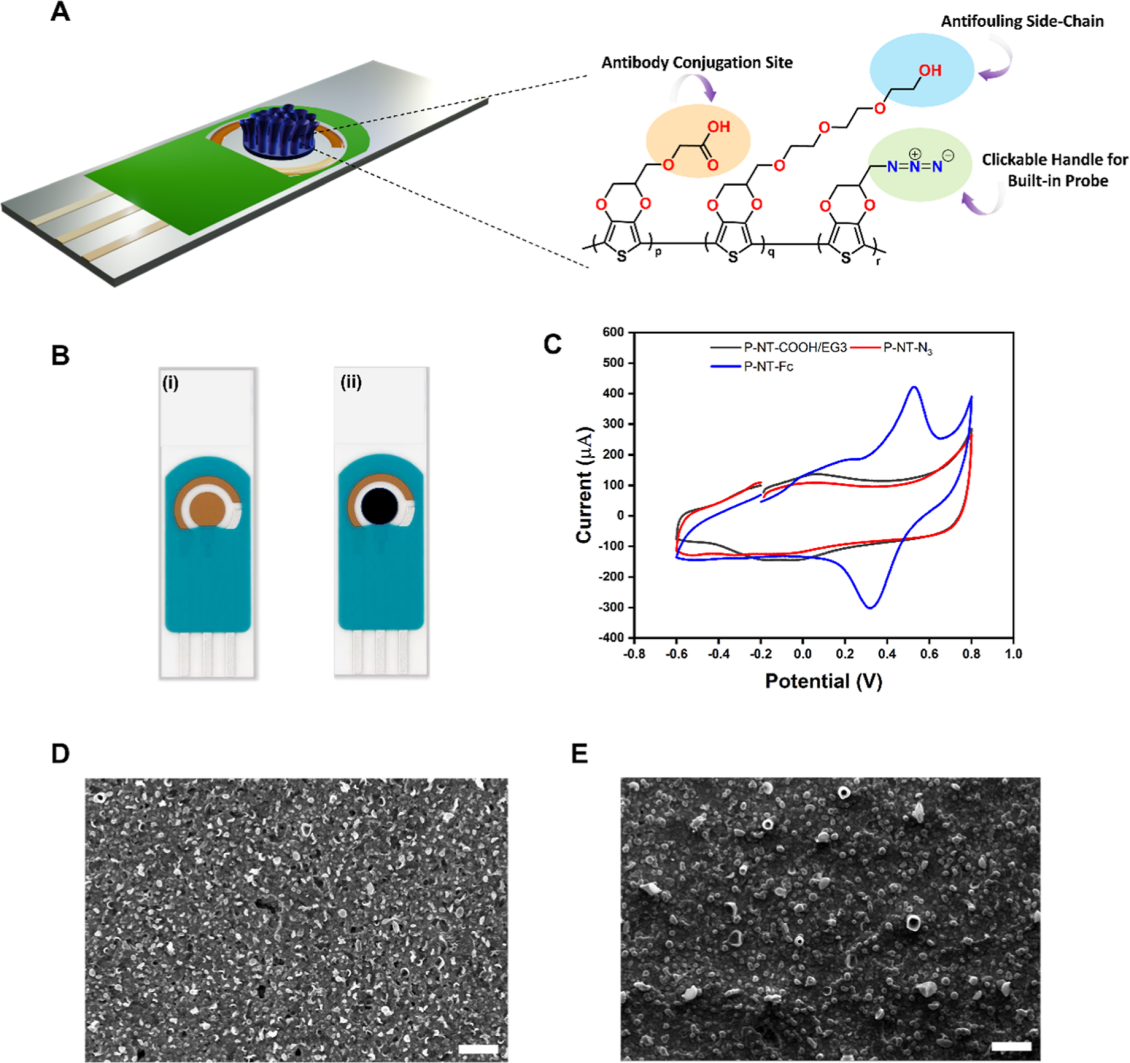
(A) The molecular design strategy to build the nano-SPE sensor platform. (B) SPE electrode platform before (i) and after (ii) electropolymerization. The SPE electrode exhibited a dark blue color after polymer deposition. (C) Cyclic Voltammograms of the SPE electrode sequentially modified with P-NT-COOH/EG3, P-NT-N_3_, and ferrocene (vs Ag). (D) Scanning Electron Microscopy (SEM) image of P-NT-COOH/EG3 nanostructures on the SPE electrode. (E) SEM image of P-NT-N_3_ nanostructures deposited on the P-NT-COOH/EG3-modified SPE electrode. Scale bar: 10 μm.

The nanostructures engineered from poly­(EDOT-COOH-*co*-EDOT-EG3) on the electrode surface were analyzed through Scanning Electron Microscopy (SEM) technique. SEM images of the polymer morphology on the Au-SPE surfaces under various electropolymerization conditions are presented in Figure S1 of the Supporting Information. Initially, a constant potential of 1.1 V was applied to electropolymerize the monomers EDOT-COOH-*co*-EDOT-EG3 for durations of 30, 60, and 90 s. When a potential of 1.1 V was applied for 30 s, we did not observe any nanostructures on the SPE electrode surface as shown in Figure S1A, instead, only a very thin polymer film was deposited on the SPE electrode surface. Increasing the time from 30 to 60 s under the same potential (1.1 V) still resulted in the formation of a polymer film without any nanostructures as shown in Figure S1B. Nanostructures with tubular morphology began to form on the SPE electrode surface when the polymerization time was increased to 90 s. However, the SEM images revealed that such nanotubes formed on the SPE surface were less dense and inhomogeneous (Figure S1C). Therefore, the applied potential was increased from 1.1 to 1.2 V while maintaining the same polymerization durations of 30, 60, and 90 s. As shown in Figure S1D, nanostructures formed at 1.2 V for 30 s were more regular and homogeneous on the SPE electrode surface compared to those formed at 1.1 V. However, the nanostructures did not sufficiently cover the electrode surface. Therefore, the polymerization time was increased to 60 s. Although we observed higher density of polymer nanostructures on the electrode surface, full electrode surface coverage was still not achieved as shown in Figure S1E. Therefore, the electropolymerization time was again increased from 60 to 90 s. Figures 3D and S1F show that high-density and homogeneous nanostructures with tubular morphology were formed on the SPE electrode surface, compared to those formed at potentials of 1.1 and 1.2 V for durations of 30 and 60 s. Additionally, a potential of 1.4 V with durations of 30, 60, and 90 s was also employed to evaluate its effect on polymer nanostructures formation on the SPE electrode surface. However, SEM analysis indicated that the electropolymerization process led to large aggregation of polymer nanostructures as shown in Figures S1G, S1H, and S1I. This phenomenon might be attributed to excessive deposition of polymer nanostructures at the higher potential of 1.4 V, leading to the aggregation of nanostructures on the electrode surface. Based on these surface morphology analysis, we concluded that the electropolymerization condition of 1.2 V for 90 s was optimal for producing the highest density, as well as regular and homogeneous poly­(EDOT-COOH-*co*-EDOT-EG3) nanotubes on the SPE electrode platform. In order to gain insight on the morphological control of nanostructure formation, we have thoroughly investigated the statistical distribution of nanotubes onto the electrode surface by measuring the diameter of different nanotubes through scanning electron microscopy (SEM) technique. The statistical distributions of nanotube diameters were investigated by SEM technique as shown in Figure S3A–C of the Supporting Information. To ensure the measurement consistency, we have measured the diameter of more than 50 nanotubes (*n* ≥ 50) from multiple locations of a single SPE electrode. We observed that, nanotubes formed on the electrode surface have diameters ranging between 500 nm–800 nm with majority of nanotubes showing diameters in the range of 600 nm–800 nm with promising density and homogeneity. The slight differences in the diameter of nanotubes formed on the electrode surface could be explained on the basis of many complex parameters that influence the kinetics of nanotube formation. The formation of nanotubes without the assistance of a well-defined template using electropolymerization technique involve highly complex kinetics interplayed from the monomer diffusion rate, nucleation density as well as the self-assembly of oligomer units etc., while these parameters are highly influenced from the applied potential, time duration, monomers stability in solvents, electropolymerization temperature as well as the electron donating/withdrawing property of functional groups tethered on the monomer side chain. In the context of nanotube formation, the solubility of radical cations formed when the potential applied along with its interaction at the electrode surface largely influences the formation of nanotubes. Our nanotube engineering process proceeds without well-defined templates, the slight differences in the nanotube’s dimensions are expected.

In order to understand the independent reproducibility of the nanotubes on the electrode surface, we have again investigated the dimensional parameters of nanotubes formed on different SPE electrodes (*n* = 3) at the identical conditions. As shown in the SEM images of Figure S3D–F, we observed that the nanotube engineering to form poly­(EDOTs) was highly reproducible as the diameter of the nanotubes from each electrode ranges from 600 nm–800 nm with very few in the range of 500 nm–600 nm as explained before. We did not observe any significant differences in the diameter of nanotubes observed on the SPE electrode. The larger tubular-like islands observed on the electrode platform might be due to the aggregation of many nanotubes formed on the electrode surface. The higher reproducibility of interelectrode fabrication process also highlights that nanotubes with 600 nm–800 nm formed as major units onto the SPE electrode surface.

The observation on the formation of nanostructures with structural parameters can be explained on the basis of charge passed through the electrode during electropolymerization, mass of polymer deposited, nucleation, and growth kinetics. We have quantified the amount of charge required to deposit the polymer on the SPE electrode surface during polymerization at different applied potentials and times. Figure S4 in the Supporting Information shows the amount of charge required to form nanotubes when poly­(EDOT-COOH-*co*-EDOT-EG3) was deposited as the first layer on the SPE electrode platform. Figure S4A–C shows the charge observed when poly­(EDOT-COOH-*co*-EDOT-EG3) was deposited at 1.1 V (vs Ag/Ag+) at 30s, 60s, and 90s, respectively. It was observed from the figure that the charge flowing through the electrode was significantly different, particularly when the applied potential was 1.1 V (vs Ag/Ag+) at 30s to 90s with significant increase in the nanotube density as seen from the SEM images in Figure S1. Similarly, the charge required to deposit the polymer was significantly higher when the applied voltage increased from 1.1 to 1.2 V (vs Ag/Ag+) as shown in Figure S4D–F. An increase in charge to deposit more polymer on the electrode surface may be due to higher nucleation and growth of the polymer through the generation of a higher number of polarons or bipolarons, which are responsible for the polymer chain growth. Such higher polymer growth eventually results in high-density nanotubes as observed by the SEM experiments. Hereafter, the first layer of polymer deposited from poly­(EDOT-COOH-*co*-EDOT-EG3) onto the SPE electrode was termed P-NT-COOH/EG3. The subsequent poly­(EDOT-N_3_) layer was termed P-NT-N_3_. After ferrocene immobilization and AFP-Ab conjugation, the modified electrodes were denoted P-NT-Fc and P-NT-Fc/AFP-Ab, respectively. The details of different poly­(EDOT) materials engineered to develop a nano-SPE sensor platform and their abbreviations used hereafter in the discussion are displayed in Table 1.

**Table 1:** Different Poly­(EDOT) Materials Engineered to Develop the Nano-SPE Sensor Platform

Poly(EDOT) material on SPE sensor	abbreviation
poly(EDOT–COOH–*co*–EDOT-EG3) Nanotube	P-NT-COOH/EG3
poly(EDOT-N_3_)	P-NT-N_3_
Ferrocene clicked poly(EDOTs) nanotube surface	P-NT-Fc
AFP-antibody conjugated poly(EDOTs) nanotube surface	P-NT-Fc/AFP-Ab

To preserve the morphology of P-NT-COOH/EG3 nanotubes on the electrode surface without damaging the nanostructures, the same potential of 1.2 V was applied to form a second layer of P-NT-N_3_ on the SPE electrode surface. As shown in the SEM images of the electrode surface morphology in Figure S2, a constant potential of 1.2 V was applied for durations of 30, 60, and 90 s. When the duration was set to 30 s, irregular and less dense nanostructures were observed on the electrode surface (Figure S2A). Thus, the electropolymerization duration was increased to 60 s. Under this condition, more homogeneous nanostructures were obtained, showing improved morphology compared to those formed at 30 s (Figures 3E and S2B). To verify whether 60 s was the best condition for forming P-NT-N_3_ nanostructures, we also tested a longer duration of 90 s. However, under these conditions, the nanostructures began to aggregate and formed small polymer islands as shown in Figure S2C, resulting in a surface morphology that was less favorable than that obtained at 60 s. Such an irregular electrode surface without significant nanostructure deposition might minimize the biosensor efficiency. Therefore, we confirmed that the condition of 1.2 V for 60 s was identified as the optimal condition for producing well-organized, high-density P-NT-N_3_ nanostructures on the SPE electrode surface based on surface morphology analysis.

The surface morphology of the second polymer layer derived from P-NT-N_3_ was further confirmed by an XPS analysis. As shown in Figure 4E, the peak observed between 400 and 405 eV indicates the presence of N 1s electrons from the azide group tethered on the PEDOT surface. The core level spectra shown in Figure S5A clearly exhibit two characteristic peaks at the N 1s region, specifically at 400 and 404 eV corresponding to the two equivalent N atoms and the one central N atom present in the azide group, respectively, along with the presence of C 1s (284 eV) and O 1s (531 eV) electrons. These observations confirm the successful formation of P-NT-N_3_ on the SPE electrode surface as shown in the Figure 4E.

**Figure 4:**
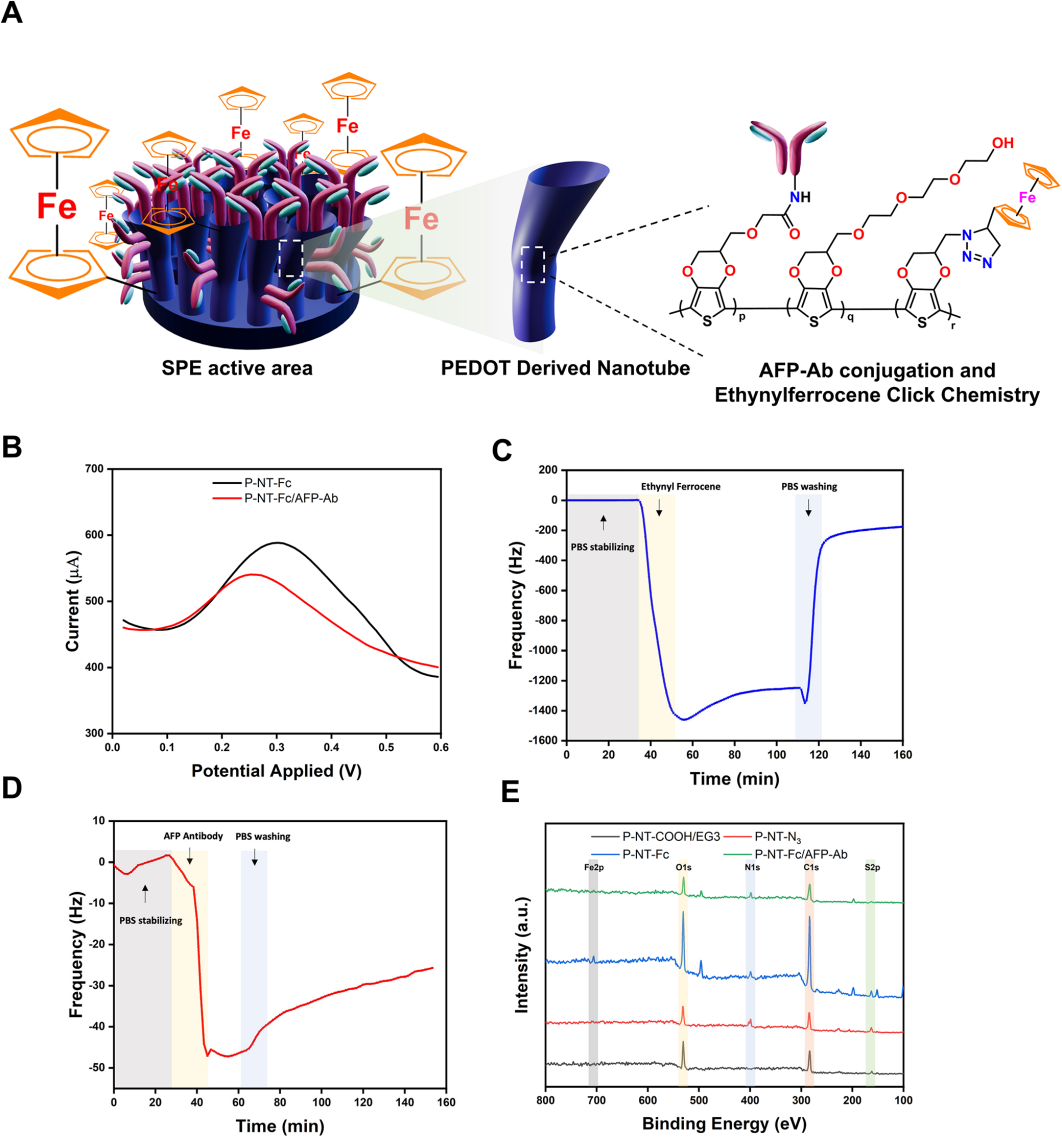
(A) Graphic illustration of the conjugation of the ferrocene-derived built-in probe and AFP-Ab on polymer nanotubes engineered on the SPE electrode. (B) DPV measurements of the SPE electrode before and after modification with ferrocene and AFP-Ab. (C) Real-time monitoring of ferrocene constructed on the P-NT-COOH/EG3 and P-NT-N_3_ nanostructured surface using QCM. (D) Real-time monitoring of AFP-Ab immobilization on the ferrocene-modified polymer nanostructured surface using QCM. A decrease in resonant frequency confirmed the successful attachment of AFP-Ab to the polymer surface. (E) XPS spectra of electrodes sequentially modified with P-NT-COOH/EG3, P-NT-N_3_, ferrocene, and AFP-Ab.

### Nano-SPE Electrode Surface Functionalization Using Ethynylferrocene through Click Chemistry

The functionalization of ferrocene on the SPE electrode surface modified with PEDOT derived nanostructures was a key step in this work toward developing a highly sensitive nanobiosensor platform. Ferrocene exhibits a distinct redox signal between 0.3 and 0.6 V which enables fast, uncomplicated electrochemical detection of the target analyte. Our idea to engineer a “build-in redox probe” derived from ferrocene to design a novel nanobiosensor platform enables an easy way to perform electrochemical detection in PBS buffer without the need for any external redox probes. In addition, the copper-catalyzed azide-alkyne click reaction is a simple and efficient strategy that does not require toxic reagents or complex experimental procedures, making it highly suitable for designing electrochemical nanobiosensors. In this study, ethynylferrocene was successfully functionalized onto the nano-SPE electrode surface using a DMF/water (2:1) solution containing ethynylferrocene (0.1M), CuSO_4_·5H_2_O (0.1M), and sodium ascorbate (0.1M) reagents through the exploration of azide-alkyne click chemistry. The successful functionalization of ferrocene was confirmed by extensive characterization techniques involving electrochemical, spectroscopic, and piezoelectric methods through CV, DPV, XPS as well as the real-time monitoring through QCM technique. Figure 3C displays the current response of the SPE electrode functionalized with ferrocene was measured in PBS buffer (1 x, pH = 7.4) at a potential ranging from −0.6 to +0.8 V. A distinct redox peak characteristic of ferrocene was observed between 0.3 and 0.6 V which was absent while measuring the redox behavior of the nano-SPE electrode modified with P-NT-COOH/EG3 as well as poly­(EDOT- N_3_) layers, as shown in the Figure 3C highlighting our successful design strategy to enable the ferrocene built-in probe onto the nano-SPE sensor platform.

This result confirmed the successful surface functionalization of the azide group presented on the electrode surface with ethynylferrocene through the copper-catalyzed 1,3-dipolar cycloaddition reaction with the formation of a stable 1,2,3-triazole ring. For a surface-confined redox system, quantitative surface coverage analysis is essential to determine the efficiency of the sensor. The surface coverage, gamma (Γ), was calculated from the integrated charge of the ferrocene redox peak from the cyclic voltammogram using the formula, Gamma (Γ) = *Q*/*nFA*, where, *Q* is the integrated peak charge values from the redox peak, *n* is the number of electrons transferred, *F* is the Faraday’s constant, and *A* is the geometrical area of the electrode platform. Since the ferrocene units were clicked on the poly­(EDOT-N_3_) layers, the integrated charge determination needs to consider the influence of capacitive charging from the conductive poly­(EDOT) under layers. Therefore, the integrated charge was calculated by integrating the anodic peak area of the ferrocene curve and divided by the scan rate. To do that, it was important to isolate the capacitive charging of the poly­(EDOTs) matrix by linear baseline fitting. The baseline was defined by the steady-state charging current observed immediately before and after the ferrocene redox peaks, and the area was integrated and divided by the scan rate to provide the net Coulombic charge associated with the surface-confined species. From the cyclic voltammogram of ferrocene as shown in the Figure S6 of the Supporting Information, the integrated charge of the surface-confined ferrocene unit on the poly­(EDOTs) layers were calculated as *Q* = 2.79851 × 10^–4^ C and the surface coverage Gamma (Γ) = 2.63678 × 10^–8^ mol/cm^2^. We were unable to find any literature data to compare the ferrocene surface coverage on the precise molecular architecture composed of nanostructured poly­(EDOTs) we designed. Therefore, a comparison was extremely difficult. Nevertheless, other literature suggested that the theoretical monolayer coverage of ferrocene on monolayer flat surfaces was in the range of 10^–10^ mol/cm^2^ to 10^–9^ mol/cm^2^.^50^ However, the surface coverage value of ferrocene units clicked on the nano-SPE electrode was much higher compared to theoretical flat monolayer surface coverage of ferrocene units. Such higher surface confinement can be explained on the basis of 3D nanostructures formed from poly­(EDOT-COOH-*co*-EDOT-EG3) and poly­(EDOT-N_3_) electrode surfaces. The SEM images have shown that the electrode surface modified with poly­(EDOT-COOH-*co*-EDOT-EG3) and poly­(EDOT-N_3_) have high-density nanotubes morphology enabling high surface area from the nanostructures. Since the azide functional groups for the click reaction were tethered on the poly­(EDOT) side chain itself, the functionalization occurred throughout the entire volume of the poly­(EDOTs) film, highlighting a bulk-loading events in the multilayer SPE electrode. Such bulk-loading of poly­(EDOTs), particularly poly­(EDOT-N_3_) at nanoscale dimension offers a higher surface area from the poly­(EDOT-N_3_) nanostructures enabling enhanced conjugation handle for the ferrocene units. In addition to that, the azide functional units were not simply sitting on the top of the nanotubes, instead the distribution occurs throughout the nanotubes on the electrode surface. Unlike self-assembled monolayer (SAM) on Au or Si surfaces, poly­(EDOT) layers decorated with nanotubes highlight a 3D-like surface ensembled with high-density nanotubes. Our previous studies highlighted the significant enhancement of binding events of different functionalities on nanostructured poly­(EDOT) surfaces compared to the surfaces without nanostructures,^37^ highlighting again the significant influence of nanostructured poly­(EDOTs) for higher conjugation of ferrocene units.

Furthermore, to support our claim on the density, surface coverage as well as the immobilization of the ferrocene redox probe on the nanotube structure of poly­(EDOTs) layers on the SPE electrode platform, we have analyzed the XPS spectra of the ferrocene conjugated SPE electrode surface. The atomic percentage of Fe atoms in the ferrocene clicked poly­(EDOT) layers is shown in Table S1 in the Supporting Information. The atomic percentage analysis of atoms obtained from the XPS analysis of the ferrocene clicked poly­(EDOTs) layers highlights a ratio of approximately 0.40 for Fe:S highlighting much high degree of functionalization, roughly for one ferrocene unit per every 2.5 thiophene unit. Such results showed successful functionalization of the poly­(EDOTs) layer with ferrocene units highlighting promising density of the redox probe on the polymer layers. Again, to visualize the distribution of ferrocene on poly­(EDOT) layers, we have performed Energy Dispersive X-ray spectroscopy (EDX) elemental mapping on the nano-SPE electrode to further support the XPS findings and confirm the spatial distribution of the ferrocene redox probes. As shown in Figure S7 in the Supporting Information, the EDX elemental mapping of Fe atoms on the ferrocene-confined nano-SPE electrode highlighted lateral homogeneity, which confirms that the “click” conjugation occurred uniformly throughout the poly­(EDOT)-derived nanotube matrix, rather than forming isolated aggregates. The combination of promising local density from XPS atomic analysis, global uniformity from EDX mapping, and excellent surface coverage values all together provides a robust physical basis for the high successful conjugation of the ferrocene probe though the click reaction on the poly­(EDOTs) layers, highlighting our novel molecular architecture design for highly efficient biosensor platforms. While a detailed investigation into the specific electrochemical mechanism and exact volumetric distribution of the ferrocene moieties on the poly­(EDOTs) matrix is beyond the scope of this study, we explored on the novel molecular design and architectural assembly at nanoscale dimensions as a strategy toward developing highly efficient biosensor platforms in the future. In addition, the successful conjugation of ferrocene was further supported by the real-time monitoring of bond formation through QCM analysis. As shown in Figure 4C, the real-time monitoring of ferrocene immobilization on the azide-modified QCM sensor chips was confirmed through the shift in resonance frequency, providing a quantitative evidence of the ferrocene molecule being successfully conjugated on the nanostructured surface decorated from functionalized PEDOTs. Initially, P-NT-COOH/EG3 and P-NT-N_3_ were electropolymerized onto the QCM sensor chip. Then, the surface was activated through CuSO_4_·5H_2_O and sodium ascorbate reagents before installing the sensor chip inside the QCM chamber. The chip was then stabilized in PBS buffer solution (1x, pH = 7.4) until the resonance frequency reached equilibrium. Subsequently, ethynylferrocene solution was introduced into the QCM sensor flow system after equilibrium was established. The drop in frequency observed at time between 30 and 40 minutes shown in Figure 4C suggests the attachment of ethynylferrocene scaffold onto the polymer surface modified with P-NT-N_3_ nanostructures. The subsequent stabilization and slight increase in frequency after washing with PBS buffer, observed after 110 minutes, further confirms that ethynylferrocene was reacted with azide functional groups on the polymer surface and successfully established conjugation on the polymer surface. Moreover, the conjugation of ferrocene on the polymer surface was also validated by XPS spectroscopy. As depicted in Figure 4E, the XPS spectrum of the surface after ferrocene modification revealed a newly emerged distinct Fe 2p signal around 710 eV. Figure S5B confirms the core level spectra of Fe 2p electrons at 707 eV and the distinctive peak highlights again the successful immobilization of Ferrocene onto the nano-SPE electrode surface.

### Immobilization of AFP-Ab through EDC/Sulfo-NHS Coupling Chemistry

Effective bioconjugation on the electrode surface is essential for the construction of high-performance immunosensors. Among various available strategies for sensor probe conjugation on electrode surfaces, we selected EDC/Sulfo-NHS coupling chemistry due to its simplicity, efficiency, and reliability for the immobilization of antibody. We successfully conjugated AFP-Ab onto the nano-SPE electrode surface decorated with PEDOT-derived nanostructures, using EDC/Sulfo-NHS coupling reagents as shown in the illustration in Figure 4A. The successful conjugation was confirmed through many analytical methods including electrochemical, spectroscopic, and piezoelectric techniques. Initially, we confirmed the successful immobilization of AFP-Ab conjugation onto the nano-SPE electrode surface through electrochemical techniques by comparing the DPV current responses before and after the AFP immobilization. Because the distinct redox peak characteristic of ferrocene appears between 0.3 and 0.6 V, the current response of the SPE electrode immobilized with AFP-Ab was measured in PBS buffer over a potential range from 0 to 0.6 V. As shown in Figure 4B, a decrease in the current was observed after the conjugation of AFP-Ab onto the nano-SPE electrode surface, confirming the successful immobilization of the antibody. Such a decrease in current might be due to the formation of an insulating layer on the electrode surface, when nonconductive AFP-Ab was attached to the P-NT-COOH/EG3 polymer layer through amide bonding. The successful conjugation of AFP-Ab on the nanostructured PEDOT-modified electrode surface was further confirmed by the real-time monitoring through QCM analysis, a versatile, powerful piezoelectric analytical tool. As shown in Figure 4D, the real-time monitoring of AFP-Ab immobilization on polymer nanostructure-modified QCM sensor chips was confirmed by a shift in resonance frequency, providing quantitative evidence that AFP-Ab was successfully conjugated to the nanostructured surface. For that study, P-NT-COOH/EG3 and P-NT-N_3_ were electropolymerized onto the QCM sensor chip, followed by immobilization of ferrocene onto the polymer surface as mentioned before. Subsequently, the carboxylic groups on the polymer surface were activated by incubating the QCM sensor chip with an EDC/Sulfo-NHS coupling reagent. Following the COOH functional group activation, the QCM sensor chip was stabilized in PBS buffer solution (1x, pH 7.4) until the resonance frequency reached equilibrium. Once a stable resonance frequency was established, AFP-Ab dissolved in PBS buffer solution (1x, pH 7.4) was introduced into the QCM flow system using the peristaltic pump. A distinct drop in resonance frequency observed at time between 30 and 40 minutes indicated the successful immobilization of AFP-Ab onto the polymer surface. The subsequent stabilization and slight increase in frequency after washing with PBS buffer, observed after 70 minutes, further confirm that AFP-Ab reacted with carboxylic functional groups on the polymer surface and successfully established conjugation on the polymer surface. Furthermore, the immobilization of AFP-Ab on the SPE electrode was also verified through an XPS analysis. As presented in Figure 4E, the XPS spectrum of the surface after AFP-Ab immobilization revealed a new peak at 399.5 eV, corresponding to the N 1s electrons originating from nitrogen atoms involved in the amide bond formation with carboxylic functional groups. The core level spectra of N 1s electrons from the amide nitrogen bonds are shown in Figure S5C. This result confirmed the successful formation of amide bonds between AFP-Ab and the carboxylic groups on the polymer surface.

### Quantitative Analysis of AFP Using the SPE Nanobiosensor Platform

The detection performance of the SPE-based nanobiosensor was evaluated by measuring the differential pulse voltammetry (DPV) response upon the binding of varying concentrations of the AFP antigen onto the SPE electrode surface. The AFP analytes interact specifically with the immobilized AFP-Ab on the SPE electrode surface, resulting in current response changes that correlate with the binding affinity between the antigen and antibody (Figure 5A).

**Figure 5:**
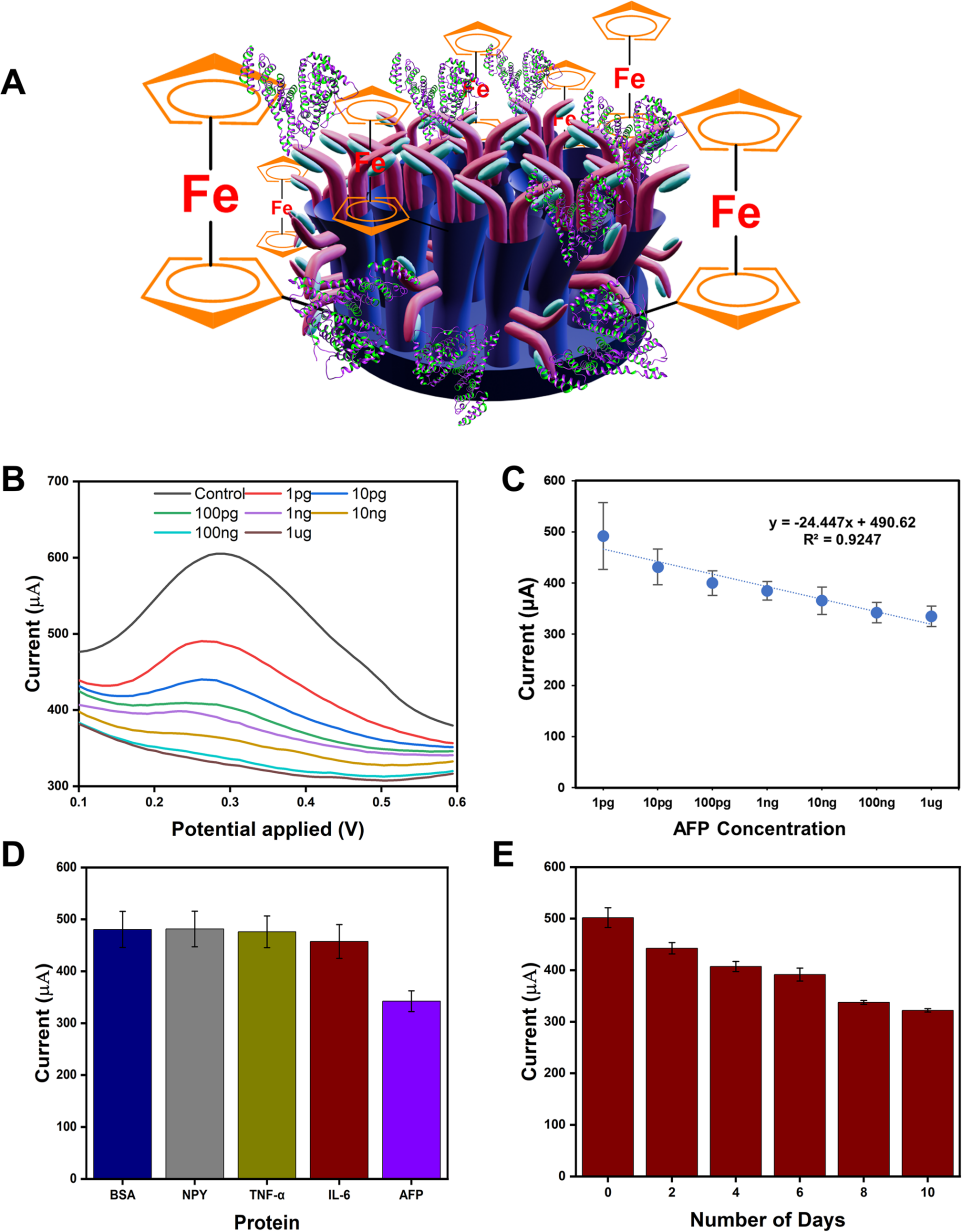
(A) Illustration showing AFP protein binding on the nano-SPE sensor platform. (B) Current responses for AFP detection using polymer nanostructure-modified SPE electrodes with AFP-Ab concentrations ranging from 1 pg/mL to 1 μg/mL (PBS, 1×, pH = 7.4). (C) Corresponding calibration curve. (D) Selectivity of the newly designed SPE nanobiosensor toward AFP in the presence of interfering compounds such as BSA (100 ng/mL), NPY (100 ng/mL), TNF-α (100 ng/mL), and IL-6 (100 ng/mL). (E) Current responses showing the shelf life performance of the SPE nanobiosensor at an AFP concentration of 10 ng/mL.

Initially, all SPE electrodes were modified with 100 μg/mL of the antibody for AFP analyte detection. AFP analyte solutions with concentrations ranging from 1 pg/mL to 1 μg/mL were prepared in PBS buffer solution (1x, pH = 7.4). As shown in Figure 5B, the electrochemical current responses corresponding to varying concentrations of AFP analytes were measured using DPV technique over an applied potential range of 0.1 to 0.6 V (vs Ag). For each measurement, the electrodes were incubated with the respective AFP analyte solution for 15 minutes. After incubation, the electrodes were washed with PBS buffer (1x, pH = 7.4) to remove any unbounded analytes. Each experiment was repeated at least 5 times to ensure the reproducibility. In Figure 5B, the electrodes exhibit distinct responses to different concentrations of AFP analyte. Notably, the current response decreased as the AFP analyte concentration increased from 1 pg/mL to 1 μg/mL. This reduction in current response with the increasing analyte concentration may be attributed to the formation of an insulating layer caused by the binding of nonconductive molecules bonded to AFP-Ab immobilized on the electrode surface. The corresponding calibration curve, derived from the linear response of current at varying analyte concentrations, is presented in Figure 5C. The calibration curve was established by recording the current response of the electrode to AFP analyte concentrations ranging from 1 pg/mL to 1 μg/mL in PBS buffer, following incubation on the electrode surface, with the corresponding standard deviations calculated for each set of measurement. The SPE nanoelectrode platform demonstrated a good linearity (*R* ^2^ = 0.9247) across a wide range of AFP analyte concentrations. This calibration curve can be expressed by the linear regression equation *y* = −24.447x + 490.62. Theoretical limit-of-detection (LOD) of the nano-SPE electrode platform was determined from the formula, LOD = 3σ/s, where σ is the standard deviation of the blank response (*n* = 5) and s is the slope of the calibration curve. The statistical value of LOD obtained using our nano-SPE electrode was 0.603 pg/mL, which is sufficient to detect the elevated concentrations of AFP in human body fluids (typically in ng/mL concentration). The analytical performance of the nano-SPE sensor for AFP detection compared to other reported AFP electrochemical sensors is listed in Table S2 of the Supporting Information.

The influence of the ferrocene density on the SPE electrode surface and the sensor sensitivity can be explained on the basis of steric hindrance and electron transfer kinetics. The key detection mechanism here for the AFP detection primarily relies on the modulation of electron transfer kinetics between the ferrocene redox probe and poly­(EDOT) layers on the electrode surface upon the binding of the AFP analyte. Such binding events were significantly influenced by the steric hindrance and electron transport mechanism. Since the signal amplitude of the ferrocene redox probe relies on the efficient transport of electrons between the poly­(EDOT) surface and the ferrocene redox probe, the number of available redox mediators (the ferrocene density) is the key factor. The increased density of the ferrocene redox probe can generally increase the sensitivity of the sensor platform through an enhanced signal amplitude. In addition to that, the electron hopping model suggests that the apparent diffusion coefficient of electrons through the ferrocene redox-active polymer is highly sensitive to the distance between redox centers. Apparently, binding of the AFP analyte on the antibody-immobilized polymer matrix can create a physical barrier, which can increase the charge-transfer resistance. Eventually, upon increasing the concentration of analyte binding on the antibody creates a larger physical barrier for electron transfer kinetics between the redox probe and polymer matrix. Insufficient availability of redox centers to communicate with the polymer matrix may decrease the sensitivity of the sensor platform due to insufficient electron transfer, which is influenced by the physical barrier created by antibody and analyte units on the polymer-coated electrode surface.

### Antifouling Effect of Poly­(EDOT-EG3) Units on the Nanoelectrode Platform

The antifouling effect of poly­(EDOT-EG3)-modified electrode platform was well studied before. Our previous studies showed that the sensor platform modified with poly­(EDOT-EG3) showed excellent antifouling properties to enhance the sensor efficiency.^51^ The antifouling effects of poly­(EDOT-EG3) functional units present in the molecular architecture of ferrocene-modified poly­(EDOT-COOH-*co*-EDOT-EG3) nanosurface were investigated by Bovine Serum Albumin (BSA) adsorption using Quartz Crystal Microbalance (QCM) technique. The QCM technique is a versatile analytical method to monitor BSA adsorbed on poly­(EDOT)- and ferrocene-modified surfaces in real-time measurements. The QCM sensor chips were modified with and without poly­(EDOT-EG3) functional units, followed by the interaction of BSA protein solution. As shown in Figure S8 in the Supporting Information, the QCM frequency was significantly dropped at 20 minutes for the control experiment (red line), while the sensor chip with poly­(EDOT-COOH-*co*-EDOT-EG3) did not show any significant drop upon BSA interaction (Figure S8, black line). These results are strongly in accordance with the previous reports and conclusions,^52,53^ highlighting that significant frequency drop in the control experiment may be attributed to the nonspecific binding of BSA proteins on the electrode platform, while the poly­(EDOT-EG3)-coated electrode exhibited excellent antifouling properties upon BSA interaction. Such molecular architectures can effectively enhance the biosensor sensitivity.

### Interference and Selectivity Analysis of the Nano- SPE Sensor Platform upon AFP Detection

In biosensor development, one of the primary challenges is achieving selective detection of target analytes in the presence of diverse interfering substances. Consequently, selectivity stands as one of the most critical performance parameters determining biosensor reliability and applicability. We evaluated the selectivity of our newly developed AFP nanobiosensor platform against common interfering compounds, including tumor necrosis factor alpha (TNF-α), Interleukin-6 (IL-6), bovine serum albumin (BSA), and neuropeptide-Y (NPY). The AFP antibody-modified SPE nanobiosensor platforms were incubated for 15 minutes with individual solutions of TNF-α (100 ng/mL in PBS), IL-6 (100 ng/mL in PBS), BSA (100 ng/mL in PBS), NPY (100 ng/mL in PBS), and AFP analyte (100 ng/mL in PBS). Following incubation, SPE electrodes were washed with PBS buffer to remove any unbound analytes. As shown in Figure 5D, the current response of the modified electrodes, detected by DPV at potentials ranging from 0 to 0.6 V (vs Ag), showed no significant change even when high concentrations of interfering substances were incubated on the surface. In contrast, a pronounced decrease in the current response was observed for the nano-SPE sensor platform incubated with AFP solution, indicating specific interaction between the AFP analyte and the SPE electrode surface. These observations highlight the successful selectivity of the SPE nanobiosensor platform.

### Shelf Life, Stability, Reproducibility, and Repeatability of the SPE Nanobiosensor

Evaluating the shelf life of the SPE nanobiosensor is crucial for determining its usable duration and long-term performance reliability and clinical practicality. The performance of nano-SPE electrodes was measured using DPV to monitor their current response over a period of time. Initially, the nano-SPE electrodes were incubated in a 10 ng/mL AFP analyte solution for 15 minutes, followed by washing with PBS buffer (1x, pH = 7.4) to remove any unbound analytes. When not in use, the electrodes were stored at 4 °C. As shown in Figure 5E, SPE electrodes exhibited an approximate 30% decrease in current response on the 10th day compared to the initial time measurements, at an applied potential ranging from 0 to 0.6 V vs (Ag). This result indicates that SPE nanobiosensor maintained acceptable long-term stability without a dramatic drop in performance over the 10 day period.

Independent fabrication reproducibility is crucial for the biosensor platform to ensure consistent, reliable, and accurate measurements. Such consistent and highly reproducible information is essential for scaling-up from benchtop to commercial, clinical, or environmental applications. We have investigated interelectrode reproducibility, intraelectrode repeatability as well as batch-to-batch variability of the nano-SPE biosensor platform using the DPV method after incubating 10 ng/mL AFP solution for 15 minutes. For the interelectrode reproducibility studies, we have fabricated four identical nano-SPE sensor platforms, followed by incubation of AFP analyte solution. The current response of all the electrodes were measured using DPV as shown in Figure 6A and each measurement was repeated minimum three times to ensure the consistence of the measurements. The peak current observed is represented as a graph chart with error bars in Figure 6D. We observed highly consistent current response with relative standard deviation (RSD) of less than 7%, highlighting a promising reproducibility of the nano-SPE sensor platforms. Similarly, we have investigated the intraelectrode repeatability of the nano-SPE electrode platform by measuring a single nano-SPE electrode fabricated by the protocol as mentioned earlier. For the intraelectrode repeatability measurements, the electrode was incubated with 10 ng/mL AFP solution for 15 minutes and washed with PBS buffer solution. The DPV measurements were recorded over a potential range from 0 to 0.6 V (vs Ag). In order to understand the intraelectrode repeatability on the interaction with AFP analyte solution, the measurements were repeated three times. The DPV curve and corresponding bar graph to show the peak currents are shown in Figure 6B and E, respectively. The DPV current measurements did not show any significant differences, highlighting a consistent repeatability of the electrode platform. Finally, the batch-to-batch variability was also investigated by fabricating independent nano-SPE electrode platforms in different batches. The different batch mentioned here is the nano-SPE electrodes fabricated at different time scales such as day-1, day-2, and day-3. Each batch, a set of nano-SPE electrode fabricated and one electrode from each batch, was incubated with 10 ng/mL AFP solution for 15 minutes. Then, each electrode was washed with PBS buffer solution to remove any unbound analytes. DPV currents were recorded by applying potential from 0 to 0.6 V (vs Ag) as mentioned before. The measurements were recorded minimum three times to ensure the consistence of measurements. The initial DPV curves and corresponding graph chart with error bars to represent the peak current values are shown in Figure 6C and F, respectively. The RSD calculated for batch-1, batch-2, and batch-3 electrode measurements were 5.16%, 3.90%, and 5.19%, respectively, highlighting excellent batch-to-batch reproducibility of the nano-SPE electrode fabrication process.

**Figure 6:**
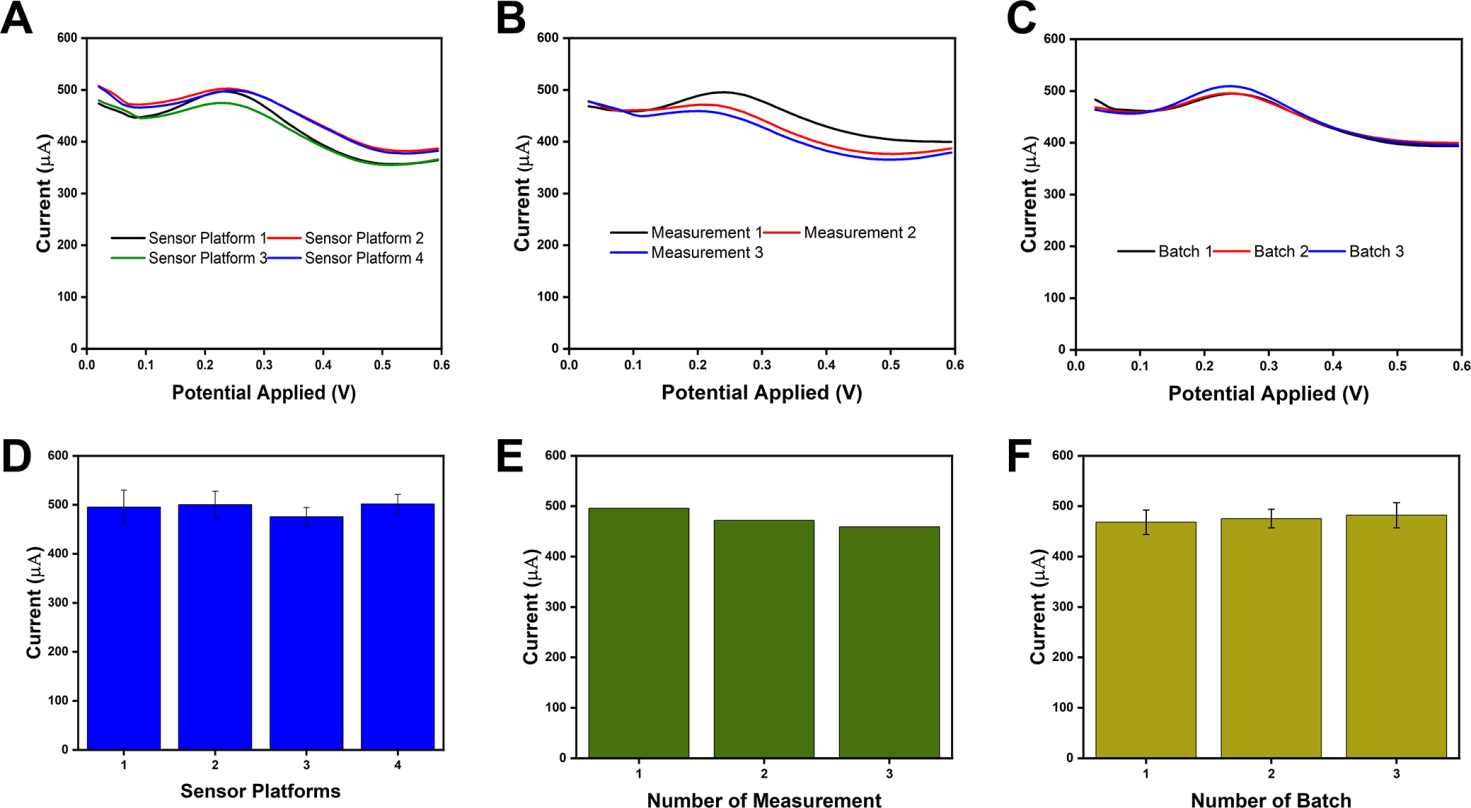
Fabrication reproducibility of the nano-SPE electrode platform. (A,D) Initial DPV current response and corresponding bar graph showing interelectrode reproducibility of four nano-SPE sensor platforms. (B, E) DPV current response of a nano-SPE electrode and corresponding bar graph for peak current showing intraelectrode repeatability. (C, F) Initial DPV current response and corresponding bar graph showing batch-to-batch variability of the nano-SPE sensor platform.

### Mechanistic Insight into Steric-Induced Analyte Detection

It is important to understand the mechanistic insights into the signal modulation and analyte binding events on a biosensor platform. The understanding of sensing events taking place on the electrode surface can provide information on various structural, chemical, or electronic parameters that influence the bioelectronic interfaces. The plausible detection mechanism behind the nano-SPE sensor platform can be explained based on the sensor architecture, nanostructure modification, and steric-induced signal modulations. The ferrocene redox probe integrated on the poly­(EDOTs) nanotubes may not be surface-confined in a flat monolayer architecture. The surface coverage analysis suggests that the ferrocene redox probe may be volume-confined within the poly­(EDOTs) nanoarchitecture. Since it was confirmed that the ferrocene redox probe was covalently appended or embedded within the nanotube matrix of poly­(EDOTs) layers, the mass transport of the ferrocene probe itself might be zero by the molecular architecture. The fixed spatial orientation within the nanotube architectures may prevent the diffusion of the ferrocene probe within the polymer matrix, allowing the signal modulation as a direct function of local environment changes instead of mass transport kinetics. Figure 7A depicts the schematic representation of a plausible sensing mechanism of the nano-SPE electrode upon the AFP analyte binding. It was proven from Figure 5B that increased binding of antigen (AFP analyte) on the nano-SPE sensor platform showed a decrease in DPV current response. Such attenuation of the current signal is possibly due to the physical shielding of the ferrocene redox centers and restrictions on ion movements for redox cycling. The AFP antibody immobilization and the antibody–antigen binding events may create a molecular crowding within the polymer lattice as shown in Figure 7A, which eventually leads to a physical barrier to cause signal attenuation. To prove the existence of such a barrier effect, we conducted a control experiment excluding the antibody conjugation by measuring the DPV currents. Figure 7B demonstrates the DPV current response before and after the incubation of 10 ng/mL AFP onto the nano-SPE sensor platform without the conjugation of the AFP antibody. It was observed from Figure 7B that the current response was similar, without any significant drop after incubating AFP analyte for 15 minutes. The results clearly highlighted that the AFP antibody was responsible for binding the AFP analyte within the poly­(EDOTs) nanotube matrix. In addition to that, Figure 4B shows a DPV current drop after AFP antibody conjugation, again proving that the immobilized ferrocene redox probe was sensitive to the surface blockage. The stepwise attenuation of the redox signal beginning with the initial conjugation of the antibody, followed by the binding of the AFP analyte, strongly supports the direct evidence of the steric-induced sensing mechanism.

**Figure 7:**
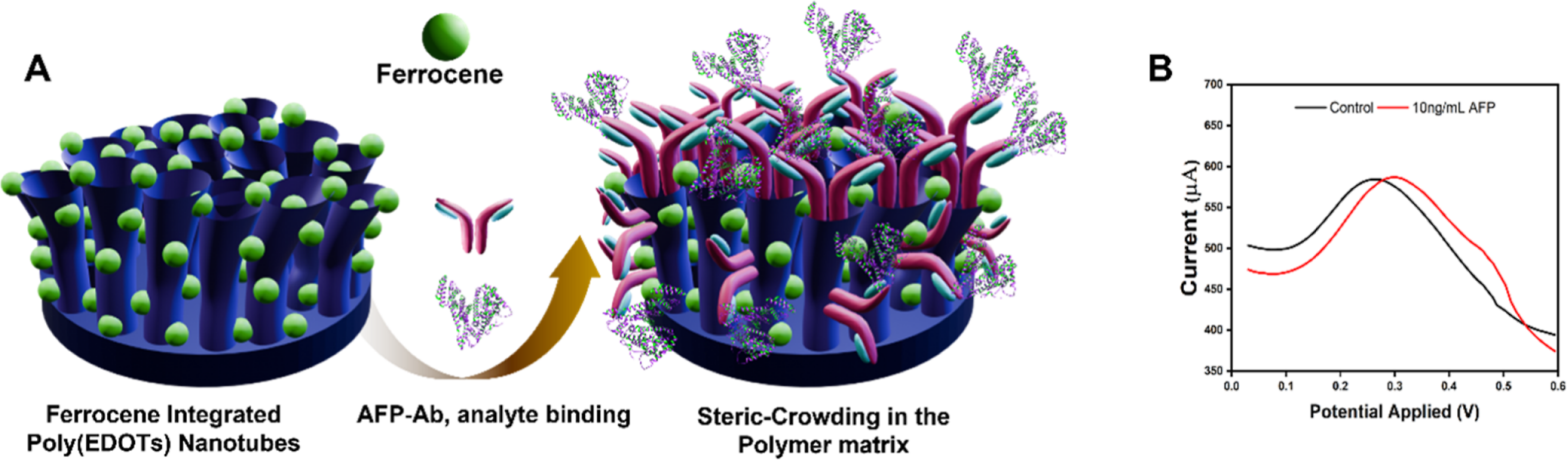
(A) Schematic representation of steric-induced crowding in the poly­(EDOTs) nanotube matrix. AFP antibody immobilization and AFP analyte binding may cause physical barrier in the ferrocene integrated nanotube matrix to induce signal attenuation. (B) DPV current measurements before and after incubating 10 ng/mL AFP analyte on the nano-SPE electrode modified with P-NT-FC without AFP antibody.

## Conclusion

In conclusion, we have designed a SPE-based nanobiosensor platform using PEDOT-derived nanostructures exploring template-free electropolymerization techniques and diverse surface chemistry strategies. The cost-effective SPE electrode was functionalized with nanostructured P-NT-COOH/EG3 and P-NT-N_3_ bilayers through template-free electropolymerization, providing functional interfaces for both bioconjugation and redox probe integration. For that, a built-in ferrocene redox probe was introduced onto the polymer nanoassemblies through ethynylferrocene-P-NT-N_3_ click chemistry while AFP-Ab was covalently immobilized via EDC/Sulfo-NHS coupling chemistry onto the P-NT-COOH/EG3 layer. In this design, poly­(EDOT-COOH) enabled efficient immobilization of AFP-Ab, poly­(EDOT-EG3) imparted antifouling properties, and P-NT-N_3_ facilitated the covalent attachment of ethynylferrocene, the built-in redox probe. The nanostructure engineering was successful through the optimization of many electrochemical parameters. The nanostructure formation from different polymer layers was confirmed through SEM technique while conjugation of the ferrocene probe and AFP-Ab were confirmed through diverse characterization techniques including QCM, XPS, CV, and DPV. The newly developed SPE nanobiosensor demonstrated promising AFP detection in PBS buffer across a wide concentration range (1 pg/mL–1 μg/mL), with a good linearity (*R* ^2^ = 0.9247) with a limit-of-detection (LOD) of 0.603 pg/mL. Moreover, the sensor exhibited excellent stability, reproducibility, repeatability, and selectivity, underscoring its potential for cancer diagnostics as a future POCT bioelectronic interface.

## Supplementary Material



## Notes

Experimental design-synthesis of monomers; optimization and surface morphology analysis of the SPE electrode with different Poly­(EDOT) nanostructures; charge calculation during nanostructure engineering; XPS core spectra of the SPE electrode after modification with poly­(EDOT-N_3_), poly­(EDOT-Fc), and AFP-Ab conjugation; surface coverage calculation of the redox probe from CV; atomic percentage and EDX mapping analysis of different polymer layers; investigation on the antifouling effect of EG3 functional units; nano-SPE sensor comparison analysis; and NMR spectra of monomers (PDF)

## Abbreviations

SPE: screen-printed electrode
AFP: alpha-fetoprotein
EDOT: 3,4-ethylenedioxythiophene
Ab: antibody
CuAAC: copper-catalyzed azide-alkyne cycloaddition
EDC: 1-ethyl-3-(3-(dimethylamino)­propyl)- carbodiimide
Sulfo-NHS: *N*-hydroxysulfosuccinimide sodium salt
Fc: ferrocene
QCM: quartz crystal microbalance
SEM: scanning electron microscopy
XPS: X-ray photoelectron spectroscopy
PBS: phosphate-buffered saline
CV: cyclic voltammetry
DPV: differential pulse voltammetry
