# Supplementary material for: Poly(3,4-ethylenedioxythiophene) Nanotube-Decorated Screen-Printed Electrodes with a Ferrocene-Derived Built-in Probe Enables Alpha-Fetoprotein Detection
Source: Langmuir. 2026 May 27;42(22):15450–65. doi: 10.1021/acs.langmuir.6c00717 (PMC13262051; doi:10.1021/acs.langmuir.6c00717)
Supplement: Supplementary file 1 [file la6c00717_si_001.pdf]

Supporting Information

Poly (3,4-ethylenedioxythiophene) Nanotube-Decorated Screen Printed Electrodes with a Ferrocene Derived Built-in Probe Enables Alpha-fetoprotein Detection

I-Chen Wang[a‡], Jayakrishnan Aerathupalathu Janardhanan[a‡], Jia-Wei She[a] and Hsiao-hua Yu*[a]

[a]Smart Organic Materials Laboratory (SOML), Institute of Chemistry, Academia Sinica, No. 128, Sec. 2, Research Institute Road, Nankang, Taipei City 115201, Taiwan.

*Corresponding author: Hsiao-hua Yu, e-mail: bruceyu@as.edu.tw.

‡I-Chen Wang and Jayakrishnan Aerathupalathu Janardhanan equally contributed to this work.

Table of Contents

1. Experimental Design-Synthesis of monomers *S3-S4*

2. QCM Experimental details of antifouling study *S4*

3.Optimization and surface morphology analysis of SPE electrode modified with poly (EDOT-COOH-co-EDOT-EG3) nanotube as first layer *S5*

4. Optimization and surface morphology analysis of SPE electrode modified with poly (EDOT-N_3) nanotubes as second layer *S6*

5. Quantitative morphological analysis of nanotubes *S7*

6. Quantitative charge analysis during nanotube engineering *S8*

7.XPS Spectra of SPE electrode after modification with poly (EDOT-N_3), poly (EDOT-Fc) and AFP-Ab *S9*

8. CV analysis for surface coverage calculation *S10*

9. Atomic percentage analysis from XPS spectra *S11*

10. Elemental Mapping from EDX Analysis *S11*

11. QCM reading on BSA adsorption and antifouling study *S12*

12.Analytical performance comparison study *S13*

13. ^{1}H NMR spectra of EDOT-OTs *S14*

14. ^{13}C NMR spectra of EDOT-OTs *S15*

15. ^{1}H NMR spectra of EDOT-N_3 *S16*

16. ^{13}C NMR spectra of EDOT-N_3 *S17*

17. References *S17-S18*

Experimental Design

Synthesis of Monomers

All the monomers used in this work were synthesized in our laboratory. The detailed synthesis of EDOT-COOH and EDOT-EG3 were reported in our previous work.[1] The EDOT-Azide (EDOT-N_3) monomer were synthesized as displayed in the scheme S1.

Part 1: Synthesis EDOT-Azide

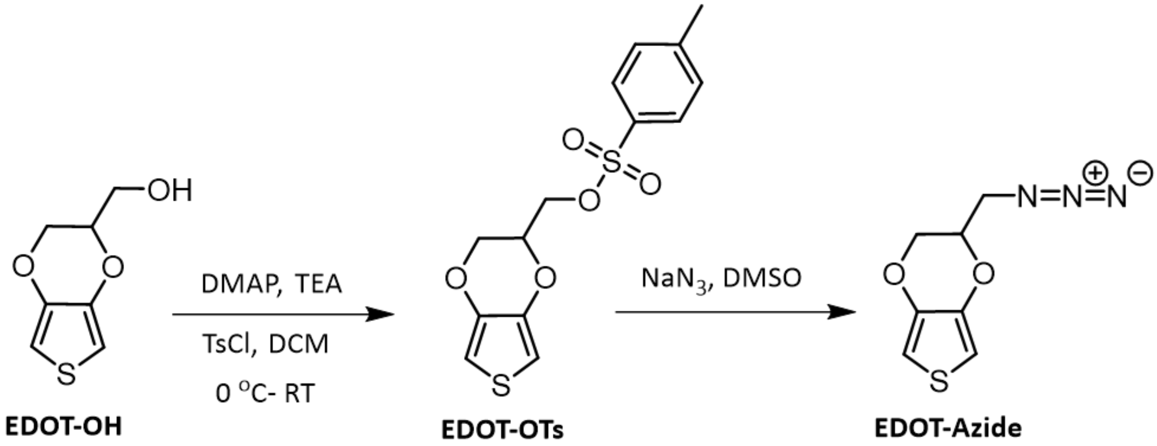

Scheme 1: Synthesis of EDOT-Azide

Synthesis of EDOT-OTs

To a solution of (2,3-dihydrothieno[3,4-b][1,4]dioxin-2-yl) methanol (EDOT-OH) (5.50 g, 31.73 mmol), TEA (5.80 ml, 57.42 mmol), and 4-dimethylaminopyridine (DMAP) (0.10 g, 0.77 mmol) in dichloromethane at 0 °C was added TsCl (8.76 g, 45.94 mmol) in small portions and the reaction was warmed to room temperature overnight. The reaction was subsequently washed with water and the organic phases combined, dried over MgSO4, and the solvent removed in vacuo. The tosylated EDOT (Ts-EDOT) was purified by column chromatography on silica with solvent changing from 1:2 to 1:0 dichloromethane:hexanes to yield 10.36 g. (82% as an oil which slowly gave a white solid under prolonged vacuum. ^{1}HNMR (400 MHz, $CDCl_3$) d 7.80 (d, J = 8.2, 2H), 7.37 (d, J = 8.2, 2H), 6.32 (d, J = 3.7, 1H), 6.26 (d, J = 3.8, 1H), 4.36 J = 6.5, 2.3, 1H), 4.20 (m or

3 overlapping dd, 3H), 4.03 (d, J = 11.6, 6.5, 1H), 2.46 (s, 3H). ^{13}C NMR (100 MHz, $CDCl_3$) δ 145.30, 140.92,140.36, 132.37, 130, 128.03, 100.23, 70.78, 66.89, 64.94, 21.69.

Synthesis of EDOT-Azide

EDOT-OTs (8.8 g,27 mmol) was added to a 250 mL round bottom flask containing 30 mL of DMSO (dimethyl sulfoxide) and 7.01g (104 mmol) of sodium azide. The reaction mixture was stirred at room temperature for 15 h. After this period of time, 15mL of dichloromethane were added to the flask. The resulting organic layer was washed with 15 mL of saturated aqueous NaCl solution. After separation, the organic layer was dried over Na_2SO4, filtered and concentrated under reduced pressure to afford product in a 91% yield (7.76 g). ^{1}H NMR (400 MHz, $CDCl_3$): δ= 6.42/6.38 (dd, 4J = 4.0 Hz, 2H, S-CH); 4.4-4.0 (m, 3H, O-CH2-CH-O); 3.7-3.5 (m, 2H, CH_2-N_3). ^{13}C NMR (100 MHz, $CDCl_3$): δ= 141.08, 140.67, 100.28, 100.12, 72.45, 65.81, 50.57

Quartz Crystal Microbalance (QCM) studies to Investigate antifouling effect

The antifouling effect of poly(EDOT-EG3) was investigated by QCM technique. For that, the QCM sensor chips were modified with poly(EDOT-COOH-co-EDOT-EG3) at +1.2 V, 90s (vs Ag/Ag^+) using 1 : 1 ratio of EDOT-COOH and EDOT-EG3 in DCM with TBAP as supporting electrolyte at 0-2 °C temperature as first layer, followed by the deposition of poly(EDOT-N_3) as second layer using 10 mM EDOT-N_3 in TBAP and DCM at room temperature at +1.2V (vs Ag/Ag^+) for 60s. The QCM sensor chip was reacted with $CuSO_4$. 5 H_2O and sodium ascorbate prior to the reaction with ethynyl ferrocene to complete the nano-sensor fabrication. For the control experiment without poly(EDOT-EG3) as antifouling agent on the electrode platform, we electropolymerized EDOT-COOH as mentioned above but used 10 mM monomer as the first layer. The deposition of poly(EDOT-N_3) and subsequent ferrocene conjugation to complete sensor fabrication protocol was the same as mentioned above. The deposition of QCM sensor chips with and without poly(EDOT-EG3) were installed in the chamber and the resonance frequency was stabilized by flowing PBS Buffer (1 x, pH = 7.4) at a flow rate of 50 µL/min. Once the frequency was stabilized, BSA protein (1 mg mL^{-1}) was run though the sensor chips at the same flow rate.

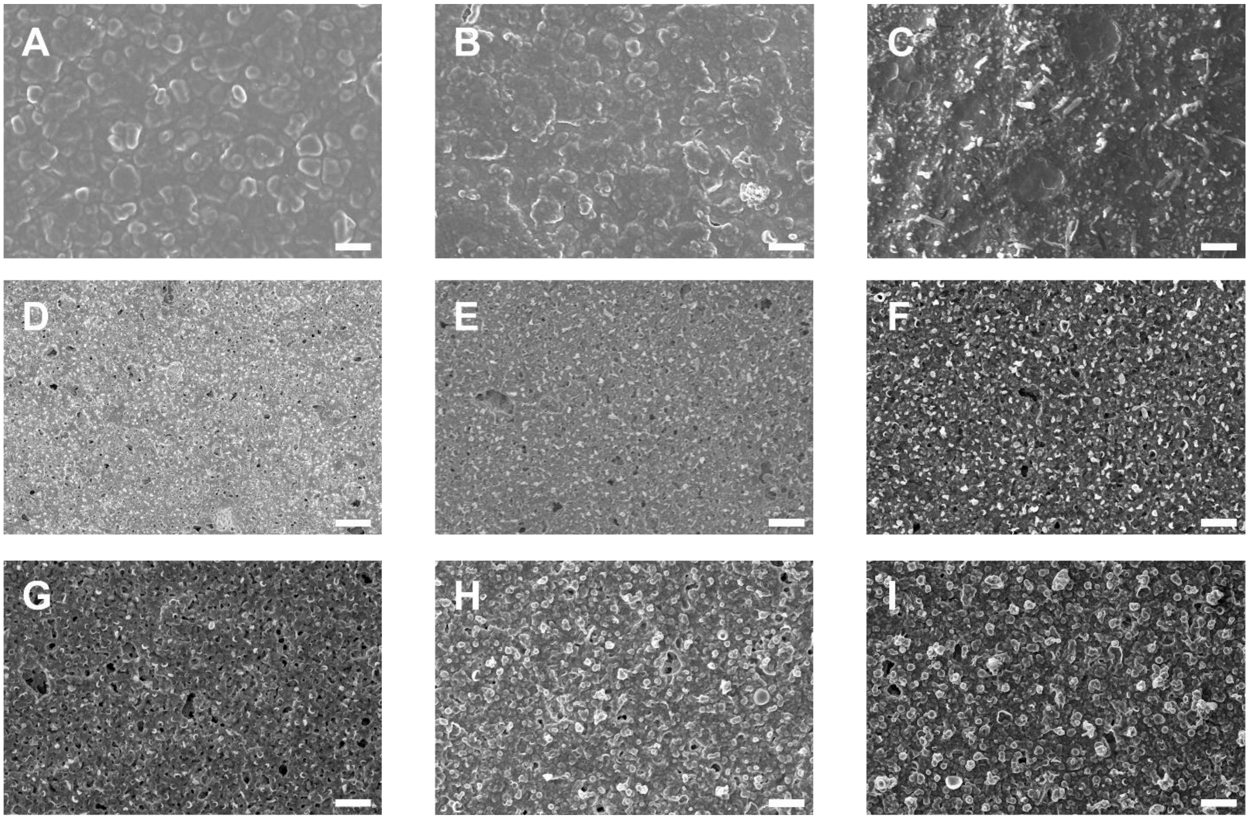

Figure S1. SEM images of SPE electrode sensor platform modified with first layer consists of EDOT-COOH–*co*–EDOT-EG3 (1:1 feed ratio) through electropolymerization at (A) 1.1 V, 30 s; (B) 1.1 V, 60 s; (C) 1.1 V, 90 s; (D) 1.2 V, 30 s; (E) 1.2 V, 60 s; (F) 1.2 V, 90 s; (G) 1.4 V, 30 s; (H) 1.4 V, 60 s; and (I) 1.4 V, 90 s in CH_2Cl_2 and tetrabutylammonium perchlorate (TBAP) as the supporting electrolyte at 0- 2 °C. Scale bars: 10 µm.

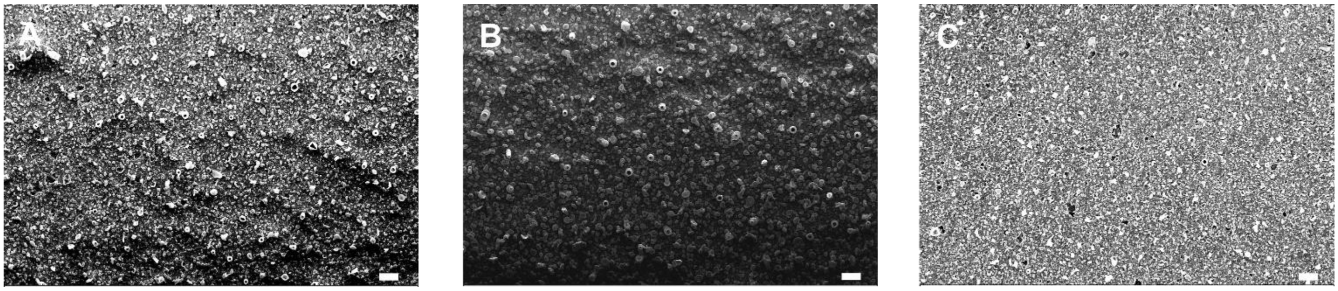

Figure S2. SEM images of nano-SPE electrode modified with second layer engineered from EDOT-azide through electropolymerization at (A) 1.2 V, 30 s; (B) 1.2 V, 60 s; (C) 1.2 V, 90 s in CH_2Cl_2 and tetrabutylammonium perchlorate (TBAP) as the supporting electrolyte at 25 °C. Scale bars: 10 µm.

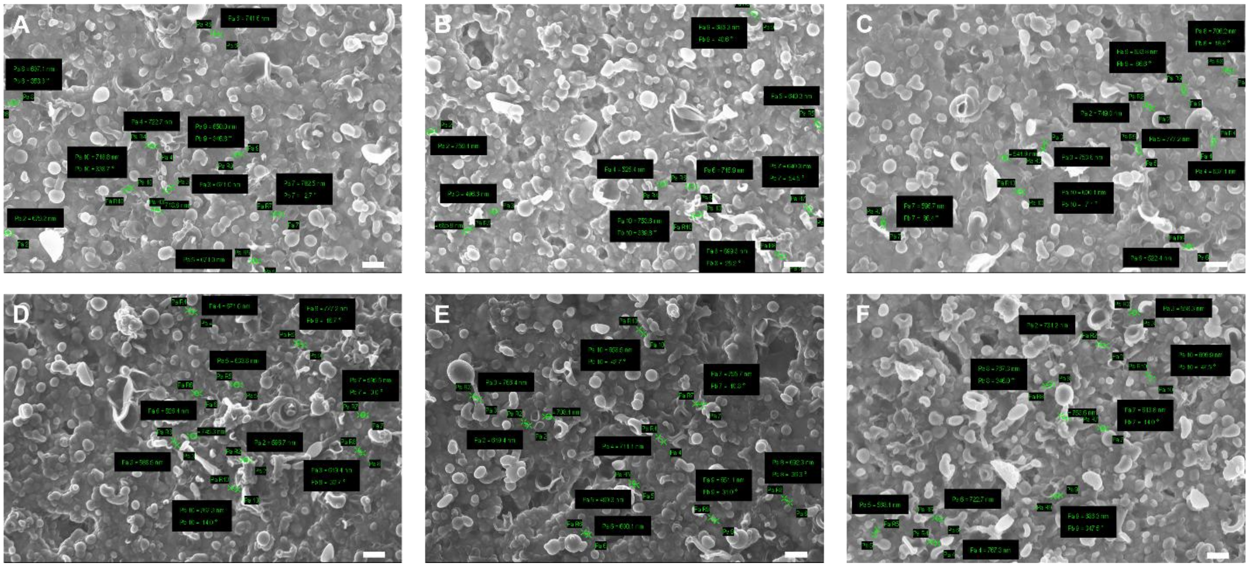

Figure S3 SEM images showing quantitative morphological analysis of nanotubes formed on the SPE electrode by template-free electropolymerization of EDOT-COOH-co-EDOT-EG3 and EDOT-N_3. SEM images A, B and C showed the dimensions of nanotubes formed at different locations of an SPE electrode area. SEM images D, E and F shown the independent fabrication reproducibility of nanotube structures on three different SPE electrodes. Scale bar 2 µm.

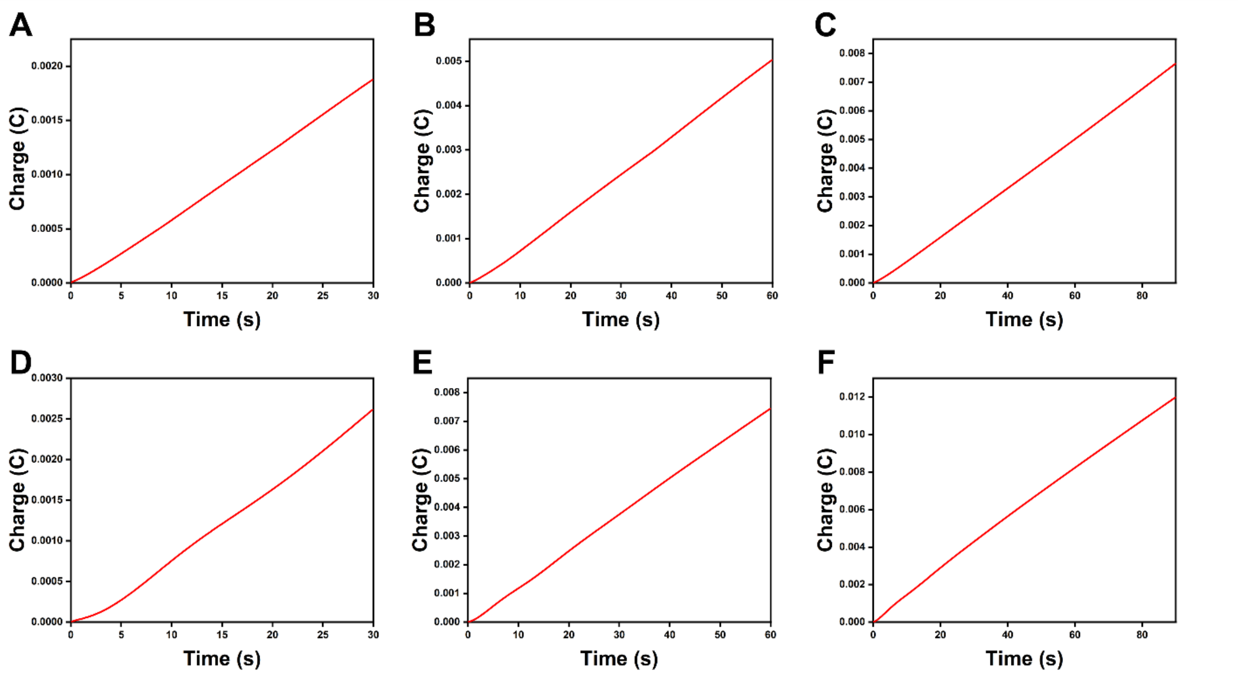

Figure S4 Electricity charge required during the electropolymerization of poly(EDOT-COOH-co-EDOT-EG3) using constant voltage method at 1.1 V (vs Ag/Ag^+) (A) 30s, (B) 60s and (C) 90s and 1.2 V (vs Ag/Ag^+) (D) 30s, (E) 60s and (F) 90s at 0-2^0 C using TBAP as supporting electrolyte in DCM solvent.

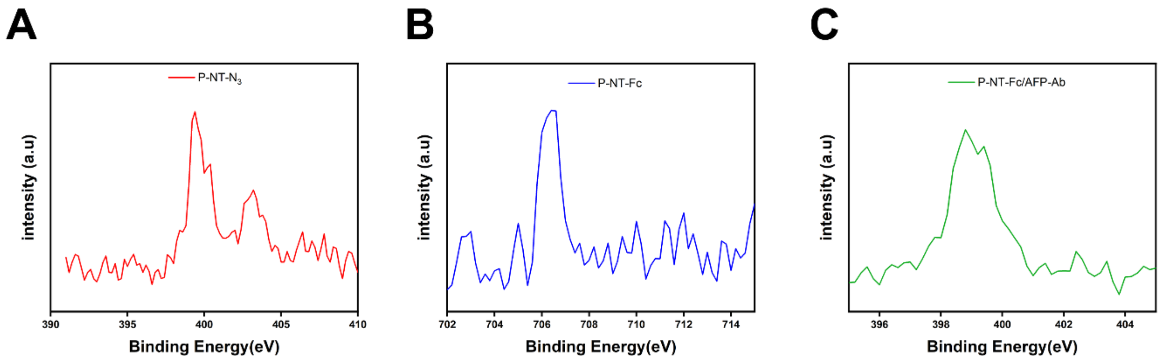

Figure S5 Core level spectra of **(A)** N 1s for P-NT-N_3 coated nano-SPE platform, **(B)** Fe 2P for Ferrocene conjugated nano-SPE platform and **(C)** N 1s for AFP-Ab conjugated nano-SPE platform.

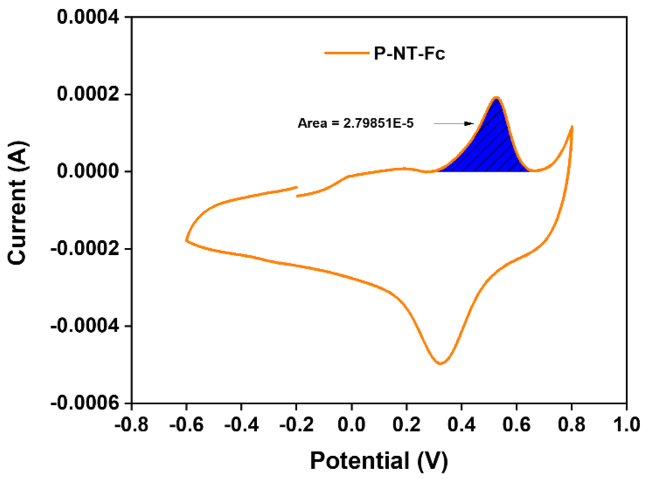

Figure S6 Cyclic voltammogram of ferrocene modified nano-SPE electrode (P-NT-FC). The integrated anodic peak area was shaded in blue color after baseline correction to eliminate capacitive charging from poly(EDOT) underlayers on the electrode.

Element	Binding Energy (eV)	Atomic Percentage (%)
C_{1s}	284	76.64
O_{1s}	531	17.21
N_{1s}	400-405	3.73
S_{2p}	165	1.73
Fe_{2p}	707	0.69

Table S1 Percentage analysis of different atoms present on the ferrocene clicked poly(EDOTs) layers on SPE electrode platform determined by X-ray photoelectron spectroscopy**.**

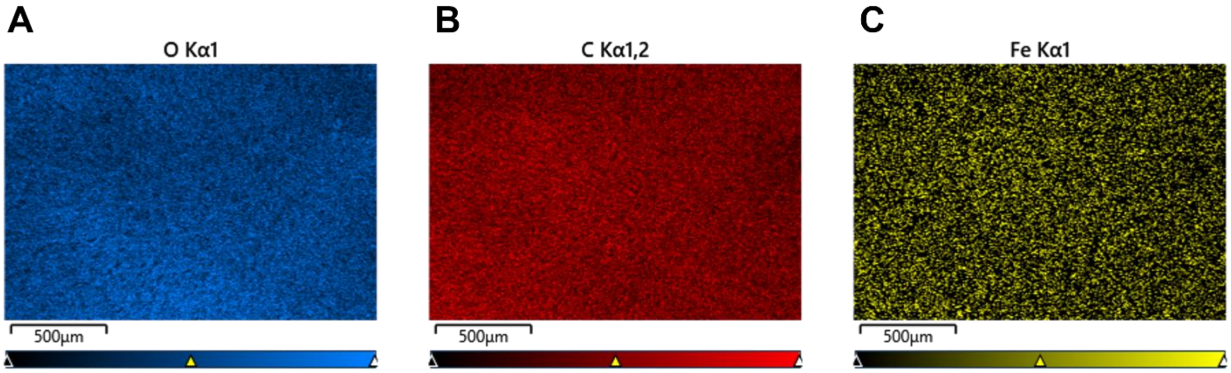

Figure S7 Elemental mapping of P-NT-Fc modified SPE electrode demonstrating uniform distribution of (A) Oxygen, (B) Carbon and (C) Iron.

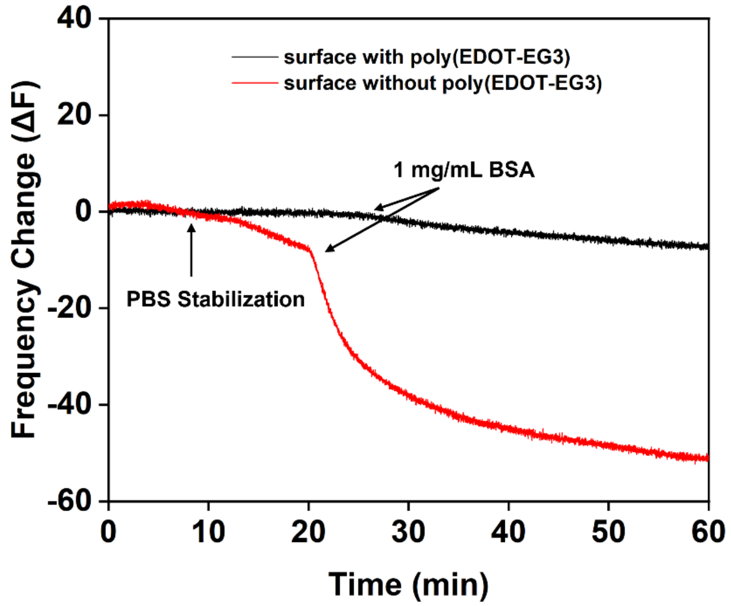

Figure S8 Real-time monitoring of anti-fouling effect exhibited by poly(EDOT-EG3) functional units. (Black line) Frequency drop (Δf) observed upon the addition of 1 mg/mL BSA in PBS on poly(EDOT-COOH-co-EDOT-EG3) and ferrocene clicked QCM sensor chip. (Red line) the control experiment without EG3 unit on QCM sensor chip upon the addition of 1 mg/mL BSA in PBS. The significant frequency drop observed on the control experiment was due to the non-specific binding of BSA protein under the absence of poly(EDOT-EG3) as antifouling agent.

Sensor Platform	Detection technique	LOD	Linear range	Ref.
PDA-APDMAO and ConA-Ag NPs	DPV	3.41 fg/mL	10 fg/mL - 10 ng/mL	2
antifouling peptide with PANI	DPV	0.03 fg/mL	0.1 fg/mL to 1 ng/mL	3
Wax-printed paper-based lateral flow device	ELISA	0.1 ng/mL	0.1ng/mL - 100 ng/mL	4
AuNP Det-Apt Conjugates biosensors	Lateral Flow	10 ng/mL	10 ng/mL to 100 ng/mL	5
Chimney-Like Fiber Optic SPR Sensor	Refractive Index	0.029 ng/mL	0-10 ng/mL	6
3-PTAA-Decorated FLG Nanosheets	CV	0.047 pg/mL	0.0001-250 ng/mL	7
Poly(EDOT-COOH-co-EDOT-EG3) nanotube with Ferrocene clicked built-in Probe	DPV	0.603 pg/mL	1 pg/mL–1 μg/mL	This work

Table S2 analytical performance of nano-SPE AFP sensor in the present work compared to other previously reported electrochemical AFP sensors.

Abbreviations

APDMAO- 3-aminopropyldimethylamine oxide; ConA-Ag NPs-concanavalin-silver nanoparticles ; PANI-polyaniline; AuNP-gold nanoparticles; SPR-Surface Plasmon Resonance; 3-PTAA -3-Polythiophene acetic acid.

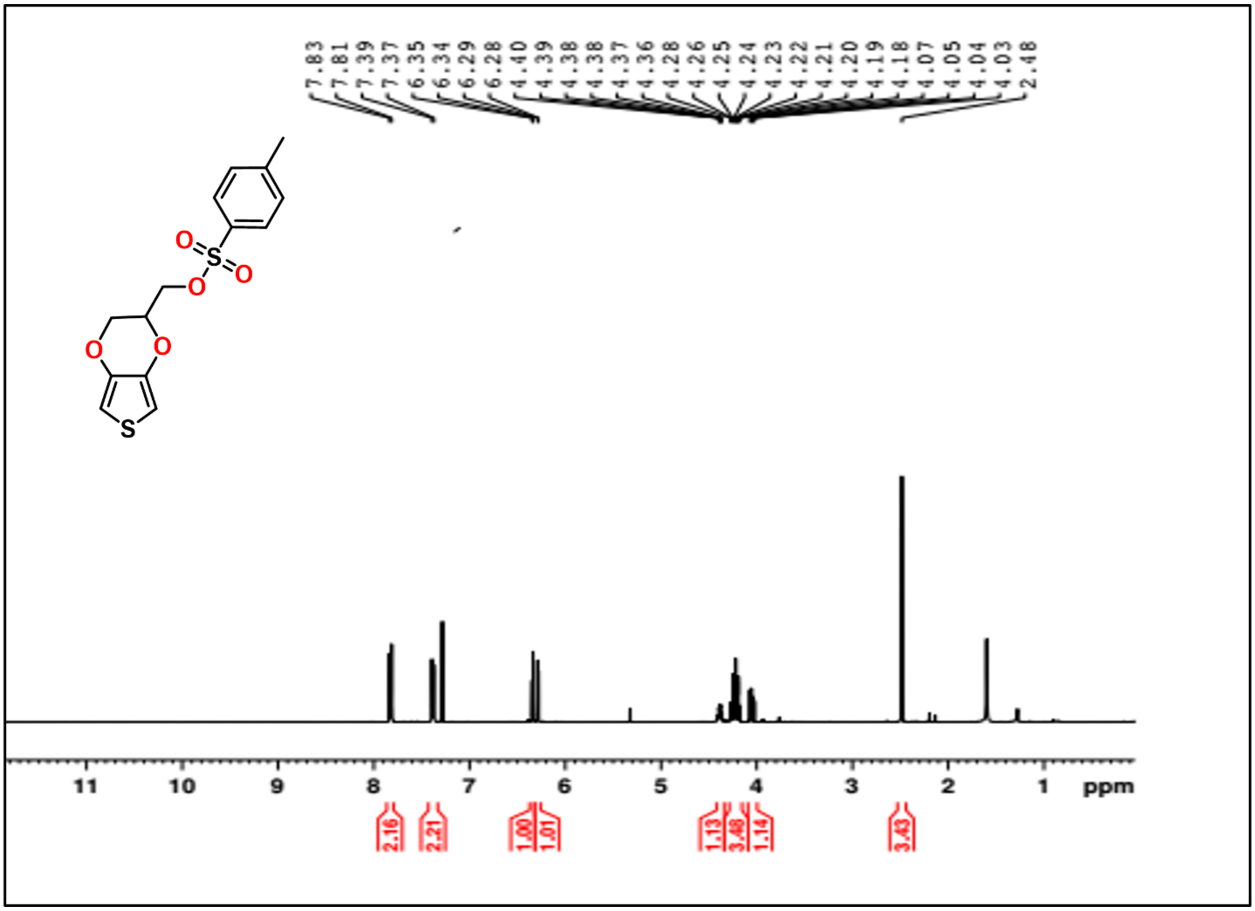

Figure S9 ^{1}H NMR Spectrum of EDOT-OTs

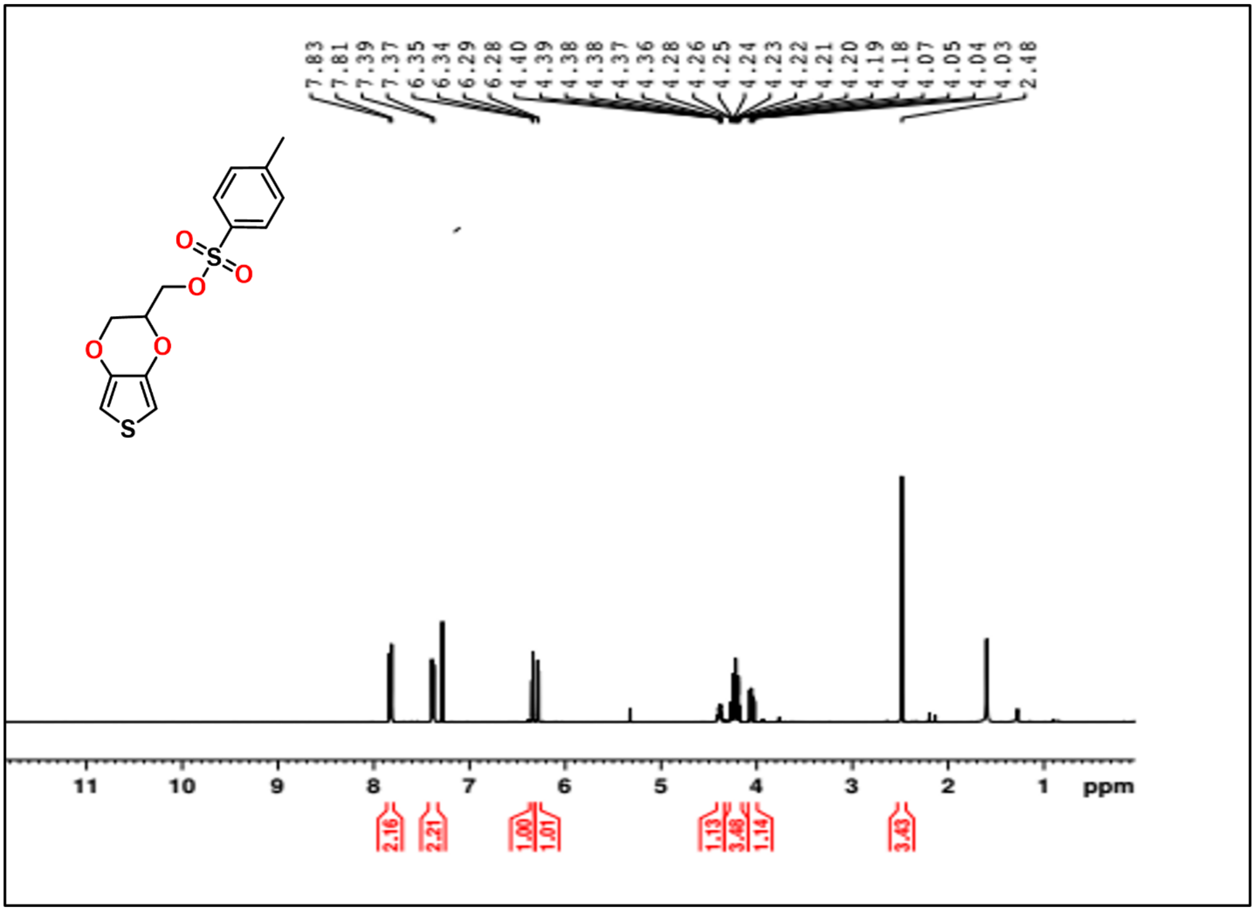

Figure S10 ^{13}C NMR Spectrum of EDOT-OTs

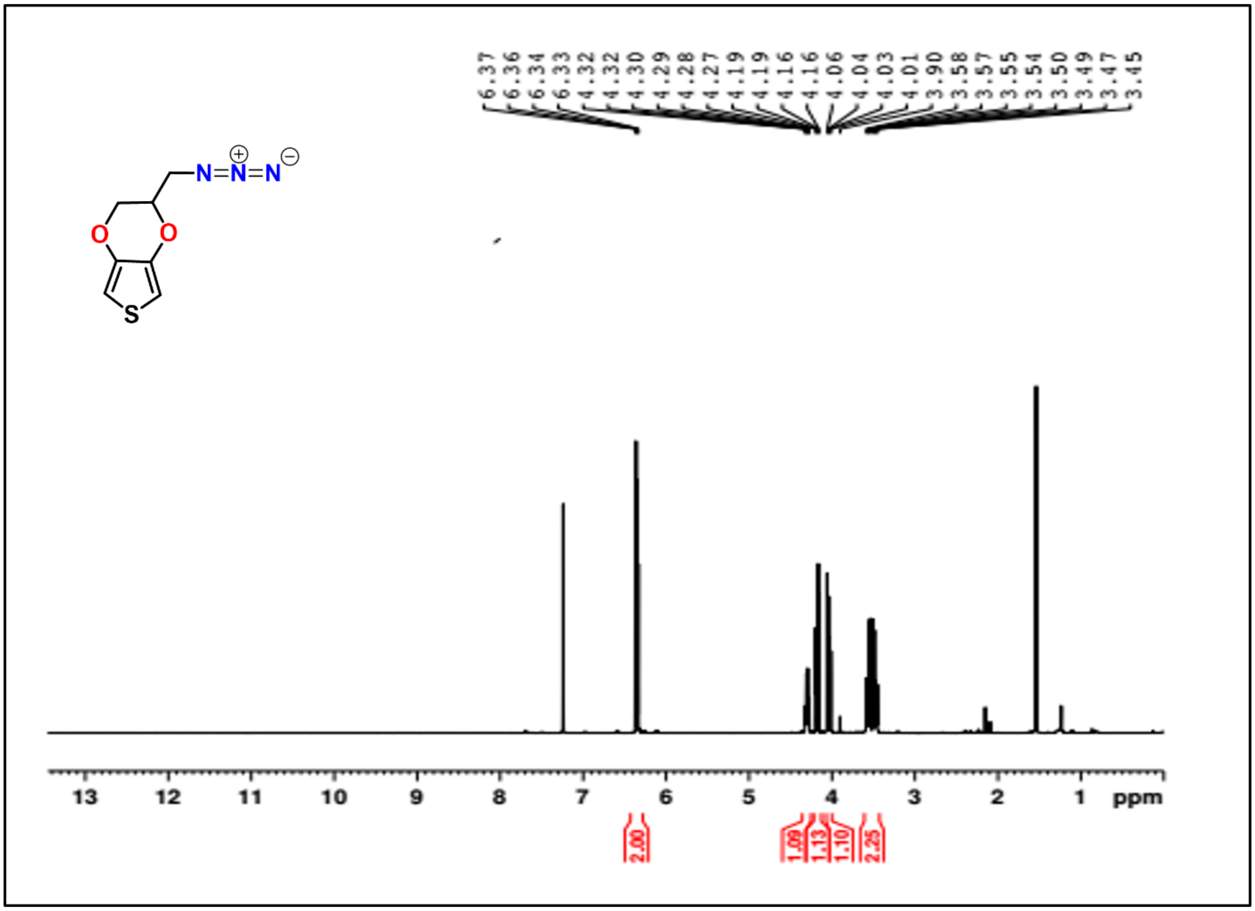

Figure S11 ^{1}H NMR Spectrum of EDOT-N_3

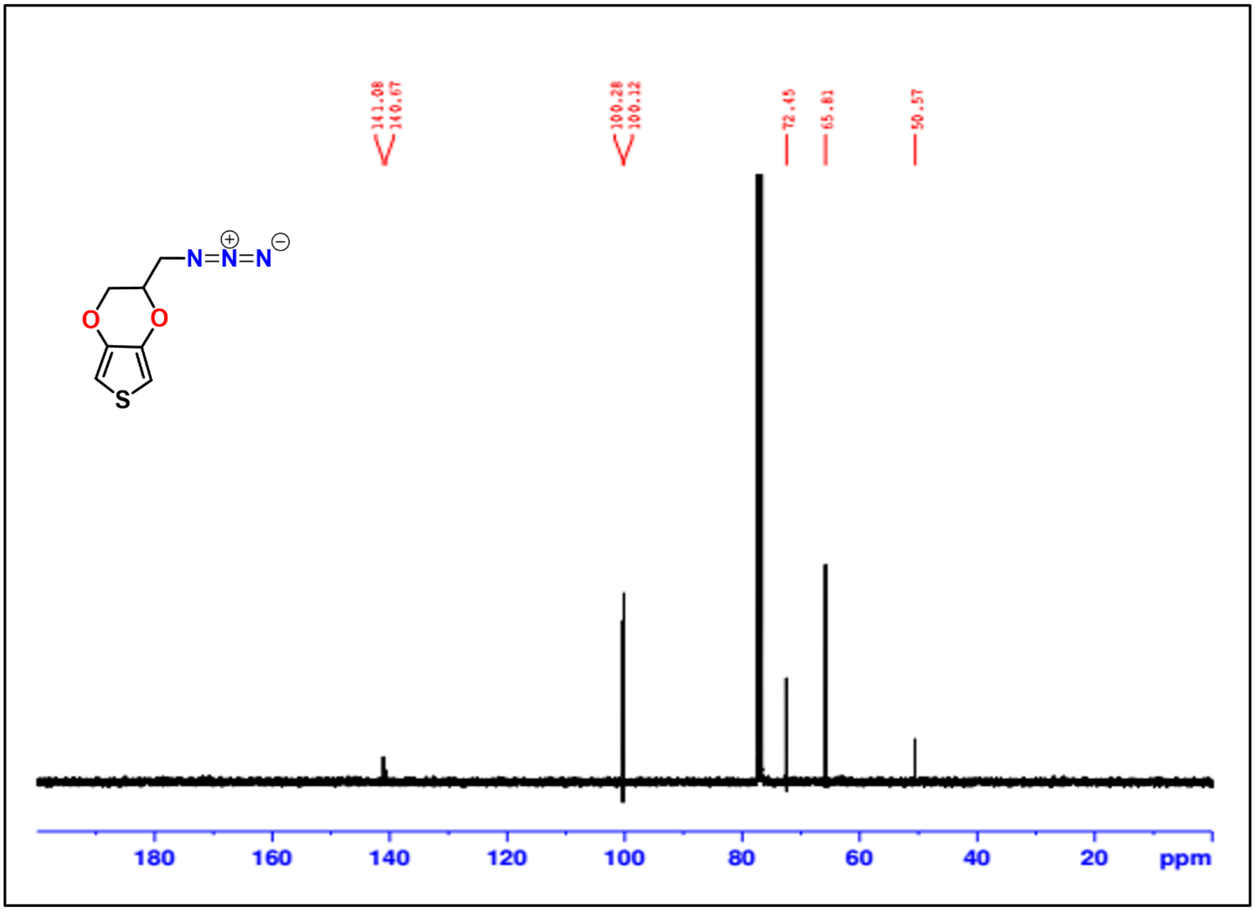

Figure S12 ^{13}C NMR Spectrum of EDOT-N_3

References

1. Aerathupalathu Janardhanan, J.; Chen, Y. L.; Liu, C. T.; Tseng, H. S.; Wu, P. I.; She, J. W.; Hsiao, Y. S.; Yu, H. H. Sensitive Detection of Sweat Cortisol Using an Organic Electrochemical Transistor Featuring Nanostructured Poly(3,4-Ethylenedioxythiophene) Derivatives in the Channel Layer. *Anal. Chem.* **2022**, *94* (21), 7584-7593.

2. Ding, Y.; Zhang, M.; Ding, M.; Ji, X.; Song, X.; Ding, C. Ultrasensitive Electrochemical Biosensor Based on Efficient PDA-APDMAO Antifouling Interface and Dual-Signal Ratio Strategy for Trace Detection of Alpha-Fetoprotein in Human Serum. *Anal Chem* **2024**, *96* (35), 14108-14115.

3. Zhao, S.; Liu, N.; Wang, W.; Xu, Z.; Wu, Y.; Luo, X. An electrochemical biosensor for alpha-fetoprotein detection in human serum based on peptides containing isomer D-Amino acids with enhanced stability and antifouling property. *Biosensors and Bioelectronics* **2021**, *190*, 113466.

4. Preechakasedkit, P.; Siangproh, W.; Khongchareonporn, N.; Ngamrojanavanich, N.; Chailapakul, O. Development of an automated wax-printed paper-based lateral flow device for alpha-fetoprotein enzyme-linked immunosorbent assay. *Biosens Bioelectron* **2018**, *102*, 27-32.

5. Ma, M.; Zhang, M.; Wang, J.; Zhou, Y.; Zhang, X.; Liu, G. Rapid Detection of Alpha-Fetoprotein (AFP) with Lateral Flow Aptasensor. *Molecules* **2025**, *30* (3).

6. Ge, C.; Peng, Y.; Shi, J.; Zhao, Y. Miniature fiber end-integrated reflective surface plasmon resonance biosensor with chimney-like structure for ultra-sensitive alpha-fetoprotein detection. *Sensors and Actuators A: Physical* **2025**, *394*, 116965.

7. Gangopadhyay, B.; Roy, A.; Paul, D.; Panda, S.; Das, B.; Karmakar, S.; Dutta, K.; Chattopadhyay, S.; Chattopadhyay, D. 3-Polythiophene Acetic Acid Nanosphere Anchored Few-Layer Graphene Nanocomposites for Label-Free Electrochemical Immunosensing of Liver Cancer Biomarker. *ACS Applied Bio Materials* **2024**, *7* (1), 485-497.
